# CD28 and CD57 define four populations with distinct phenotypic properties within human CD8^+^ T cells

**DOI:** 10.1002/eji.201948362

**Published:** 2019-12-04

**Authors:** Luca Pangrazzi, Jürgen Reidla, José Antonio Carmona Arana, Erin Naismith, Carina Miggitsch, Andreas Meryk, Michael Keller, Adelheid Alma Nora Krause, Franz Leonard Melzer, Klemens Trieb, Michael Schirmer, Beatrix Grubeck‐Loebenstein, Birgit Weinberger

**Affiliations:** ^1^ Department of Immunology Institute for Biomedical Aging Research University of Innsbruck Rennweg 10 Innsbruck Austria; ^2^ Department of Internal Medicine Clinic II Medical University of Innsbruck Anichstrasse 35 6020 Innsbruck Austria; ^3^ Department of Orthopedic Surgery Hospital Wels‐Grieskirchen Grieskirchnerstrasse 42 Wels Austria

**Keywords:** CD8+ T cells, CD28, CD57, cell differentiation, vaccination

## Abstract

After repeated antigen exposure, both memory and terminally differentiated cells can be generated within CD8^+^ T cells. Although, during their differentiation, activated CD8^+^ T cells may first lose CD28, and CD28^−^ cells may eventually express CD57 as a subsequent step, a population of CD28^+^CD57^+^(DP) CD8^+^ T cells can be identified in the peripheral blood. How this population is distinct from CD28^−^CD57^−^(DN) CD8^+^ T cells, and from the better characterized non‐activated/early‐activated CD28^+^CD57^−^ and senescent‐like CD28^−^CD57^+^ CD8^+^ T cell subsets is currently unknown. Here, RNA expression of the four CD8^+^ T cell subsets isolated from human PBMCs was analyzed using microarrays. DN cells were more similar to “early” highly differentiated cells, with decreased TNF and IFN‐γ production, impaired DNA damage response and apoptosis. Conversely, increased apoptosis and expression of cytokines, co‐inhibitory, and chemokine receptors were found in DP cells. Higher levels of DP CD8^+^ T cells were observed 7 days after Hepatitis B vaccination, and decreased levels of DP cells were found in rheumatoid arthritis patients. More DP and DN CD8^+^ T cells were present in the bone marrow, in comparison with PBMCs. In summary, our results indicate that DP and DN cells are distinct CD8^+^ T cell subsets displaying defined properties.

## Graphical abstract

Through microarray analysis and functional assays, we investigated the phenotype of CD28+CD57‐, CD28+CD57+, CD28‐CD57‐ and CD28‐CD57+ CD8+ T cells in humans. Our study provides new insights into T cell differentiation and senescence. Furthermore, our work suggests the importance of CD28+CD57+ CD8+ T cells in the context of vaccination and diseases.

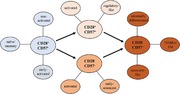

## Introduction

Persistent antigenic stimulation supports the accumulation of late differentiated T cells, particularly within the CD8 compartment 1. To achieve complete activation of naïve and memory CD8^+^ T cells, a costimulatory signal is required in addition to the first signal provided by the interaction of the TCR with the complex MHC/peptide 2. The best‐defined co‐stimulus is represented by the interaction between the co‐stimulatory receptor CD28 expressed by T cells and its ligands CD86 and CD80 on the surface of APCs 3.

After their activation, CD8^+^ T cells differentiate into cytotoxic T lymphocytes (CTL) and memory cells, which can be maintained for long periods of time in tissues such as the BM 4, 5, 6. After persistent antigenic stimulation and several activation cycles, CD28 expression is progressively downregulated on the surface of CD8^+^ T cells 3, 7. CD8^+^CD28^−^ T cells are characterized by short telomere length, reduced antigen‐induced proliferation and enhanced production of pro‐inflammatory molecules 8, 9, 10, 11. In addition, this population has been shown to be associated with reduced immune response to pathogens and vaccines in old age and increased mortality in the elderly 12, 13. Alongside the down‐regulation of CD28, CD8^+^ T cells gain the expression of the terminal differentiation marker CD57 14. It has been suggested that, after repeated contact with antigens, activated CD28^+^CD57^−^ CD8^+^ T cells may first reduce the surface expression of CD28, therefore becoming CD28^−^CD57^−^, and then express CD57, consequently acquiring the phenotype of terminally differentiated CD28^−^CD57^+^ CD8^+^ T cells (reviewed by Strioga et al. 15). Despite this theory, a population of CD28^+^CD57^+^ CD8^+^ T cells has also been identified in the peripheral blood (PB), indicating that the linear CD28/CD57 CD8^+^ T cell differentiation model may be more complex than previously thought 14, 16. In addition, it has been suggested that CD8^+^ T cell terminal differentiation may only be dependent on CD57 14.

In this study, we investigated the phenotype of CD28^+^CD57^+^ (DP) and CD28^−^CD57^−^ (DN) CD8^+^ T cells, in comparison to the already well‐characterized CD28^+^CD57^−^ and CD28^−^CD57^+^ CD8^+^ T cell populations. While DN cells expressed lower levels of TNF and IFN‐γ in comparison to the other subsets, the expression of CD8^+^ T cell effector molecules was similar to CD28^−^CD57^+^ cells. DP CD8^+^ T cells were characterized by increased cytokine production, in particular IL‐10, which was higher in comparison to the other three subsets. In addition, the levels of HLA‐DR, activation molecule 4‐1BB, and co‐inhibitory molecules PD‐1, CTLA‐4, and TIGIT, were higher in the DP subset. Chemokine receptors, such as CCR5 and CXCR5, were overexpressed by DP CD8^+^ T cells in comparison to all the other subpopulations. When purified subsets were cultured and stimulated with αCD3 and IL‐2, both the DP and DN CD8^+^ T cell subsets could be generated from CD28^+^CD57^−^ cells. Seven days after a booster vaccination against HBV, the frequency of DP CD8^+^ T cells, as well as CXCR5^+^ DP CD8^+^ T cells, increased in comparison to day 0, and afterward dropped back to pre‐vaccination levels. Decreased levels of DP cells and increased frequency of CXCR5^+^DP CD8^+^ T cells were found in rheumatoid arthritis patients in comparison to age‐matched controls. Thus, using the markers CD28 and CD57, four subsets with distinct and unique properties can be defined within CD8^+^ T cells. These findings may help in understanding more about how immune responses take place and the mechanisms governing the differentiation from naïve to memory and to terminally differentiated T cells.

## Results

### CD28^+^CD57^+^ and CD28^−^CD57^−^CD8^+^ T cells display unique phenotypes

**Figure 1:**
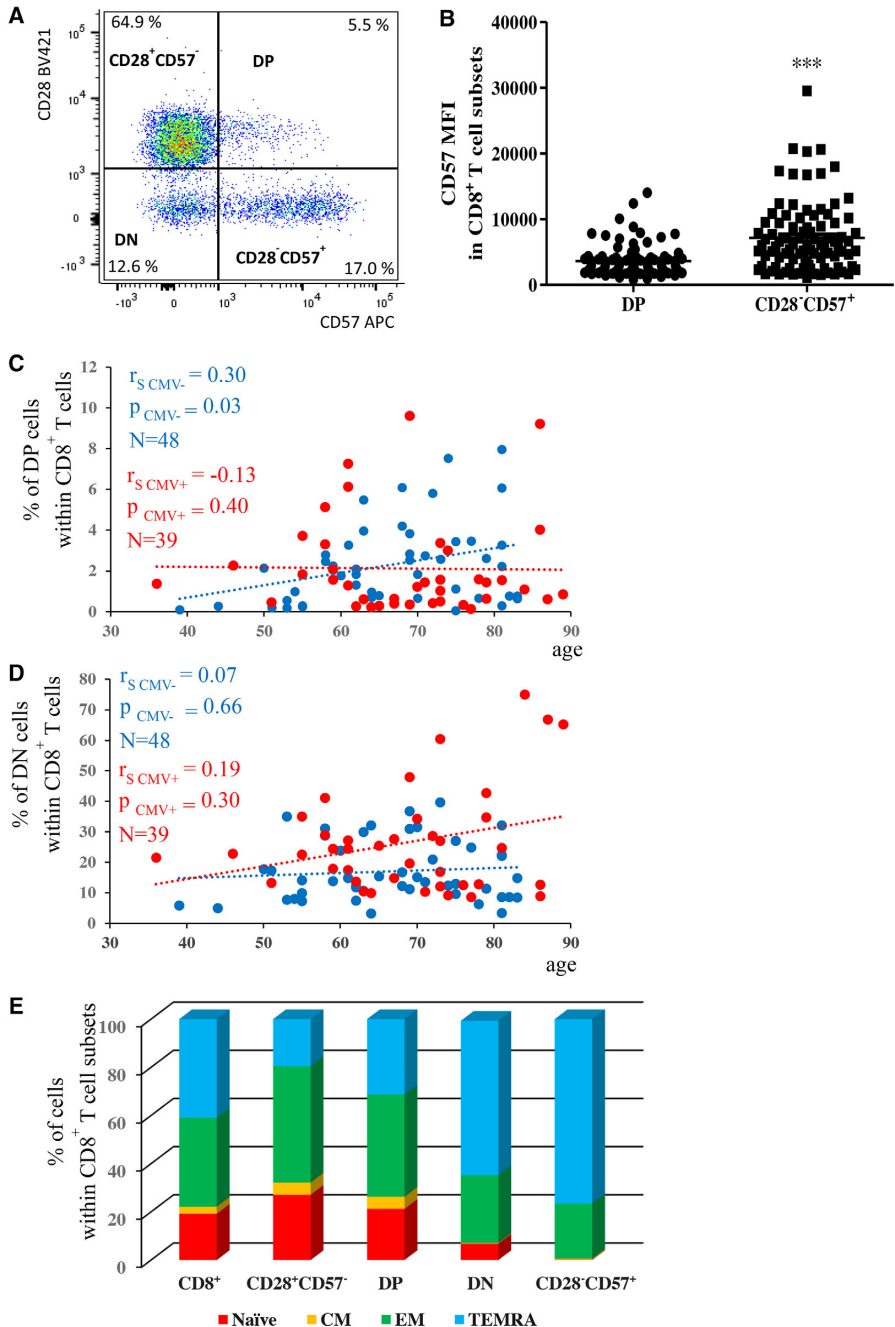
Phenotype of CD28^+^CD57^−^, DP, DN, and CD28^−^CD57^−^CD8^+^ T cells. (A) Representative flow cytometry plot showing gating strategy for CD28^+^CD57^−^, DP, DN, and CD28^−^CD57^+^ populations within CD8^+^ T cells in PBMCs obtained from healthy donors. (B) MFI of CD57 in DP and CD28^−^CD57^+^CD8^+^ T cells. Paired *t*‐test, *N* = 91, ***p < 0.001. Samples were pooled from 91 independent experiments with one sample per experiment. Correlation of the frequency of DP (C) and DN (D) cells within CD8^+^ T cells and age in CMV^−^ (blue) and CMV^+^ (orange) donors. In each graph, data are shown as mean ± SEM and pooled from 91 independent experiments with one sample per experiment. Spearman coefficient (r_s_), sample number (*N*), and *p*‐values are reported in the figure. (E) Diagram showing the mean frequency (*N* = 91) of naïve, CM, EM, and TEMRA (defined using the markers CCR7 and CD45RA) within CD8^+^, CD28^+^CD57^−^CD8^+^, DP CD8^+^, DN CD8^+^, and CD28^−^CD57^+^CD8^+^ T cells.

Using the markers CD28 and CD57, populations of CD28^+^CD57^−^, CD28^+^CD57^+^(DP), CD28^−^CD57^−^(DN), and CD28^−^CD57^+^ cells were identified within CD8^+^ T cells in PBMCs. A representative plot of the gated populations is shown in Figure 1A. Interestingly, while DP cells showed an intermediate expression of CD57, CD28^−^CD57^+^ CD8^+^ T cells were both CD57^dim^ and CD57^high^. As a consequence, CD57 MFI was higher in CD28^−^CD57^+^ compared to DP cells (Fig. 1B). CD28^+^CD57^−^ CD8^+^ T cells decrease with age whereas CD28^−^CD57^+^ CD8^+^ T cells increase (data not shown), corroborating earlier reports 15, 16, 17 and results in our group 18. Whether age also affects the frequencies of DP and DN CD8^+^ T cells is currently unknown. Therefore, we measured the percentages of DP and DN cells within CD8^+^ T cells in PBMCs in CMV seropositive and seronegative donors of different age (Fig. 1C and D). Frequencies of DP CD8^+^ T cells increased in CMV^−^ but not in CMV^+^ persons (Figure 1C). No significant correlations were found between frequency of DN CD8^+^ T cells and age in both CMV^+^ and CMV^−^ persons (Figure 1C).

We next investigated the distribution of CCR7^+^CD45RA^+^ naïve, CCR7^+^CD45RA^−^ CM, CCR7^−^CD45RA^−^ EM, and CCR7^−^CD45RA^+^ TEMRA subpopulations within DP CD8^+^ T cells, in comparison to the DN, CD28^+^CD57^−^, and CD28^−^CD57^+^ CD8^+^ T cell subsets and the whole CD8^+^ T cell population (Fig. 1E; Supporting Information Fig. 1). CD28^+^CD57^−^ and DP cells, similarly to CD8^+^ T cells, were distributed in all, naïve, CM, EM, and TEMRA subpopulations, with increased frequencies within the naïve, CM, and EM subsets (Fig. 1E; Supporting Information Fig. 1A–C). DN and CD28^−^CD57^+^ CD8^+^ T cells showed a similar distribution and were mainly included within the EM and TEMRA subsets, but not in the naïve and CM subpopulations (Fig. 1E; Supporting Information Fig. 1D and E). As expected, the highest frequency of cells within TEMRA and the lowest levels of CM and naïve were found within CD28^−^CD57^+^ CD8^+^ T cells.

To further characterize the phenotype of the four cell subsets, gene expression profiles of FACS sorted CD28^+^CD57^−^, DP, DN, and CD28^−^CD57^+^CD8^+^ T cells were studied using Affymetrix arrays. Gene Set Enrichment Analysis (GSEA) was performed in order to identify pathways that are differentially regulated between the four subpopulations. A summary of the pathways significantly enriched after GSEA in the comparisons between the populations are reported in Supporting Information Figure 2. Expression of genes coding for proteins involved in IFN‐γ responses and G2/M checkpoint control increased both from CD28^+^CD57^−^ to DP and from CD28^+^CD57^−^ to DN cells. Mitotic spindle genes were overexpressed in DN but not in DP cells when compared to CD28^+^CD57^−^ cells. Furthermore, expression of MYC targets was reduced both from CD28^+^CD57^−^ to DP and from DN to CD28^−^CD57^+^ cells, while DNA repair genes were downregulated from CD28^+^CD57^−^ to DP cells and IL‐2 STAT‐5 signaling genes from DP to CD28^−^CD57^+^ cells, respectively. These results suggest that CD28^+^CD57^−^, DP, DN, and CD28^−^CD57^+^ CD8^+^ T cells show distinct phenotypes.

### DP and DN CD8^+^ T cells show differential expression of cytotoxic and effector molecules

CD8^+^ T cells are known to overexpress pro‐inflammatory molecules when they become terminally differentiated and/or senescent 11, 12. Additionally, reduced levels of CD28 and increased expression of CD57 have been indicated as markers for highly differentiated T cells. In order to assess the stage of T cell differentiation in DP and DN cells in comparison to CD28^+^CD57^−^ and CD28^−^CD57^+^ cells, we investigated the expression of cytotoxic molecules and markers for highly differentiated T cells in CD28^+^CD57^−^, DP, DN, and CD28^−^CD57^+^ CD8^+^ T cell subsets, first analyzing the data collected using microarrays. The gene expression of selected molecules involved in cytotoxic and terminal differentiation features in the four subsets is reported in Table 1. Overall, genes encoding for pro‐inflammatory and cytotoxic molecules, NK markers as well as transcription factors supporting CD8^+^ T cell terminal differentiation progressively increased from CD28^+^CD57^−^ to CD28^−^CD57^+^ subsets. Indeed, the expression of most of these genes was lowest in CD28^+^CD57^−^ cells, intermediate in DP, slightly higher in DN, and the highest was in CD28^−^CD57^+^ cells (Table 1). The chemokine receptor CX3CR1 has been shown to reflect the degree of CD8^+^ T cell differentiation 19. While the expression of CX3CR1 was low in the CD28^+^CD57^−^ subset, the levels of this transcript progressively increased in DP, DN, and CD28^−^CD57^+^ cells (Table 1). These results were confirmed at the mRNA level using qPCR (data not shown). The transcription factor Hobit (ZNF 683) has been shown to support the expression of granzyme B in highly differentiated T cells 20. Again, ZNF 683 expression was lowest in CD28^+^CD57^−^, intermediate in DP, significantly higher in DN compared to DP, and the highest in the CD28^−^CD57^+^ subset (Table 1). Similar results were observed for the expression of granzyme B, granzyme H, perforin 1, and the cytotoxic molecule FGFBP2 (Table 1). Expression of NK markers increase in highly differentiated T cells 21. Indeed, most of NK receptors were low in CD28^+^CD57^−^ cells, intermediate in DP, and higher in DN CD8^+^ T cells (Table 1). No differences were found between the DN and CD28^−^CD57^+^ subsets.

**Table 1:** Genes coding for proteins involved in cytotoxic and effector functions in CD28^+^CD57^−^, DP, DN, and CD28^−^CD57^+^CD8^+^ T cells

	CD28^+^ CD57^−^	DP	DN	CD28^−^ CD57^+^	DP versus CD28^+^CD57^+^	DP versus DN	DP versus CD28^−^CD57^+^	DN versus CD28^+^CD57^−^	DN versus CD28^−^CD57^+^
CMKLR1	4.71	7.34	11.04	14.09	***	***	***	***	***
PROK2	5.38	7.37	8.14	11.87	*	ns	***	***	***
CX3CR1	6.34	10.88	12.42	14.16	***	*	***	***	***
CCL4	7.06	11.12	11.03	11.87	**	ns	ns	***	ns
ZNF683	8.15	8.37	11.44	12.91	ns	***	***	***	*
TBX21	6.11	7.87	9.27	9.80	***	***	***	***	ns
FGFBP2	10.18	14.03	16.05	17.12	***	***	***	***	***
GSAP	6.60	6.73	8.57	8.53	ns	ns	*	***	ns
GZMB	7.32	9.81	11.76	13.70	***	***	***	***	***
GZMH	10.50	13.85	14.28	15.20	***	***	***	***	***
PRSS23	4.87	4.92	5.21	6.51	ns	ns	***	ns	**
NUAK1	4.17	4.32	4.73	7.45	ns	ns	***	***	***
FCGR3A	4.39	7.14	10.61	11.95	***	***	***	***	ns
FCGR3B	4.52	7.00	9.74	11.12	***	***	***	***	***
SH2D1B	4.47	5.98	11.74	10.09	**	***	***	***	ns
NCR1	5.26	6.85	10.44	10.11	*	***	***	***	ns
KLRC3	9.95	10.38	13.78	12.75	ns	***	*	***	ns
KLRD1	11.23	13.23	15.01	15.43	***	***	***	***	ns
KLRF1	8.82	10.82	12.95	13.05	***	***	***	***	ns
KLRC3	9.95	10.38	13.78	12.75	ns	***	*	***	ns
KLRG1	12.47	14.21	14.22	14.45	***	ns	ns	***	ns
KLRC2	10.82	10.59	13.96	12.85	ns	***	***	***	ns
KIR3DX1	4.67	4.75	7.43	7.74	ns	***	***	***	ns
KIR2DS5	5.43	6.87	10.44	9.67	*	***	***	***	ns

The relative gene expression of each gene measured using microarrays is reported. For each individual gene, lower expression is shown in blue, higher expression in red, and intermediate levels are reported in purple. One‐way ANOVA, Bonferroni post hoc test. *p < 0.05, **p < 0.01, ***p < 0.001. *N* = 4 for each subset. Data are combined from one representative experiment.

**Figure 2:**
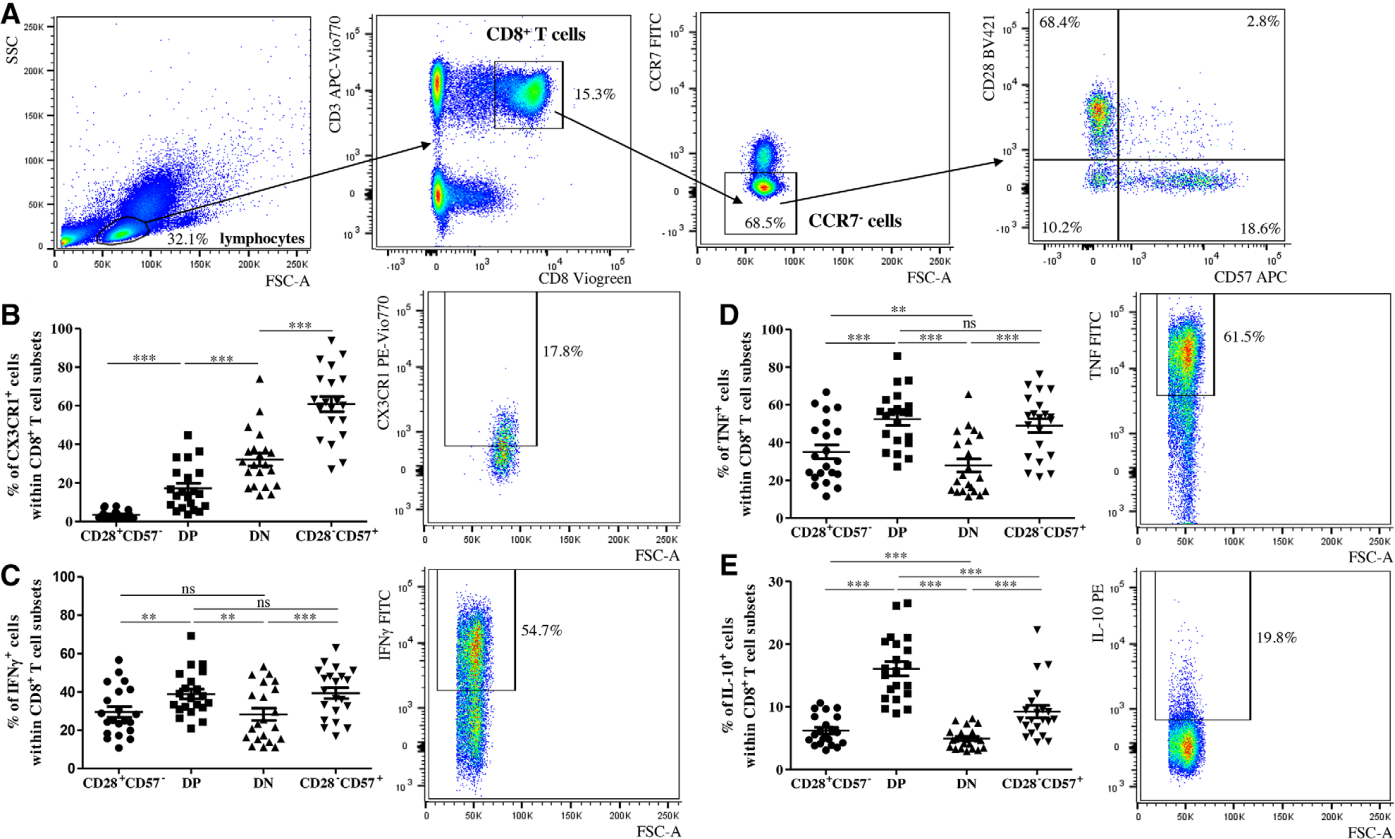
Cytotoxic and effector molecules in CD28^+^CD57^−^, DP, DN, and CD28^−^CD57^+^ cells within CCR7^−^CD8^+^ T cells. (A) Complete gating strategy used to define the populations of interest in the experiments performed using flow cytometry. After gating on CD3^+^CD8^+^ T cells within lymphocytes, effector (CCR7^−^) cells and the CD28^+^CD57^−^, DP, DN, and CD28^−^CD57^+^ subsets were defined. Frequency of CX3CR1^+^ (B), TNF^+^ (C), IFN‐γ^+^ (D), and IL‐10^+^ (E) cells within CD28^+^CD57^−^, DP, DN, and CD28^−^CD57^+^ CCR7^−^CD8^+^ T cells in PBMCs obtained from healthy donors. Representative flow cytometry plots reporting the expression of each molecule within the DP subset are shown. N = 21 for each graph. Data are shown as mean ± SEM and pooled from 14 independent experiments. One‐way ANOVA, Bonferroni post hoc test. *p < 0.05, **p < 0.01, ***p < 0.001.

In order to reduce the heterogeneity within the four subsets, the protein expression of T cell effector molecules was assessed using flow cytometry in the same populations after gating on CCR7^−^ (effector) CD8^+^ T cells (Figure 2). The complete gating strategy is shown in Figure 2A. As observed for the mRNA, CX3CR1 levels were low in the CD28^+^CD57^−^ subset, intermediate in DP, slightly more expressed by DN and highly expressed by CD28^−^CD57^+^ CD8^+^ T cells (Figure 2B). Similar results were observed for the four populations within CD8^+^ T cells without gating on CCR7^−^ cells first, although no differences were found between the CD28^+^CD57^−^ and DP subsets (Supporting Information Fig. 3A). We next investigated the production of CD8^+^ T cell cytokines, TNF, IFN‐γ, and IL‐10 after stimulation with PMA, ionomycin in the presence of BFA in CD28^+^CD57^−^, DP, DN, and CD28^−^CD57^+^ CCR7^−^CD8^+^ T cells using flow cytometry (Fig. 2B–D). TNF was lower in the DN population, intermediate in CD28^+^CD57^−^ and it was the highest in both DP and CD28^−^CD57^+^ CD8^+^ T cells (Fig. 2C). The frequency of IFN‐γ^+^ cells was low in both the CD28^+^CD57^−^ and DN subsets and higher in DP and CD28^−^CD57^+^ CD8^+^ T cells (Fig. 2D). Interestingly, increased percentages of IL‐10^+^ cells were found in DP CD8^+^ T cells in comparison to the other subsets (Fig. 2E). The same results were found when the expression of the cytokines was measured in the whole CD8^+^ T cell population (Supporting Information Fig. 3B–D)

These findings indicate that DN cells may be phenotypically distinct from DP CD8^+^ T cells. In particular, the two subsets differ substantially in terms of production of cytokines and effector molecules.

### Expression of surface molecules involved in T cell function differ between DP and DN CD8^+^ T cells

**Figure 3:**
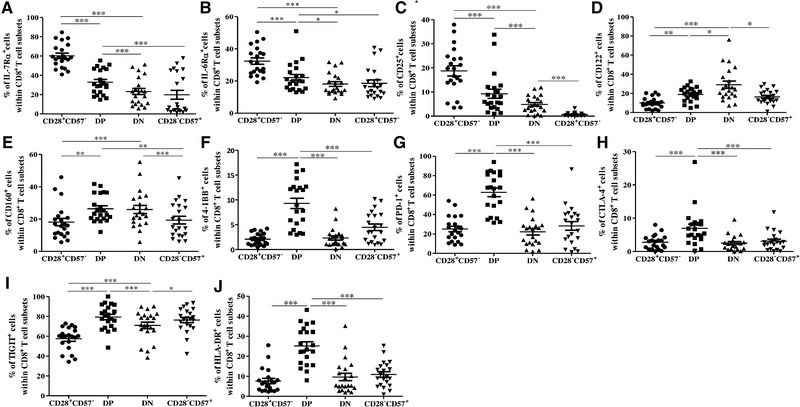
Expression of cytokine receptors, activation and co‐inhibitory molecules in CD28^+^CD57^−^, DP, DN, and CD28^−^CD57^+^ cells within CCR7^−^CD8^+^ T cells. Frequency of IL‐7Rα^+^ (A), IL‐6Rα^+^ (B), CD25^+^ (C), CD122^+^ (D), CD160^+^ (E), 4‐1BB^+^ (F), PD‐1^+^ (G), CTLA‐4^+^ (H), TIGIT^+^ (I), and HLA‐DR^+^ (J) cells within CD28^+^CD57^−^, DP, DN, and CD28^−^CD57^+^ CCR7^−^CD8^+^ T cells measured using flow cytometry in PBMCs obtained from healthy donors. *N* = 21 for each group. One‐way ANOVA, Bonferroni post hoc test. *p < 0.05; **p < 0.01; ***p < 0.001. Data are shown as mean ± SEM and pooled from 14 independent experiments.

T cell differentiation affects the expression of cytokine receptors, as well as activation and co‐inhibitory molecules on T cells. To learn more about the responsiveness to T cell cytokines, we measured the levels of the cytokine receptors IL‐7Rα, IL‐6Rα, CD25, and IL‐2/IL‐15Rβ (CD122) in DP and DN cells, in comparison to CD28^+^CD57^−^ and CD28^−^CD57^+^ CD8^+^ T cells (Fig. 3A–D; Supporting Information Fig. 4A–D). Again, the expression of the molecules of interest was measured within effector CCR7^−^CD8^+^ T cells. Overall, the expression of IL‐7Rα, IL‐6Rα, and CD25 was reduced from CD28^+^CD57^−^ to the DP subset. Lower levels of the three receptors were found in DN compared to DP cells. In CD28^−^CD57^+^ CD8^+^ T cells, levels of IL‐7Rα and IL‐6Rα were similar while the expression of CD25 was reduced in comparison to the DN subset. In contrast to the other cytokine receptors, levels of CD122 were the highest in DN compared to the other CD8^+^ T cell subsets, suggesting that increased expression of this receptor may somehow compensate the reduced levels of IL‐7Rα, IL‐6Rα, and CD25 in DN cells (Fig. 3D). Very similar results were obtained when the four receptors were measured in the whole CD8^+^ T cells, without gating on CCR7^−^ cells (Supporting Information Fig. 5A–D).

We then assessed the expression of markers of activation, in particular the activation receptor CD160 and the inducible co‐stimulatory receptor 4‐1BB (CD137) in the four CD8^+^ T cell subsets (Fig. 3E and F; Supporting Information Fig. 4E and F). CD160 levels increased from CD28^+^CD57^−^ to DP and they did not change between DP and DN cells, indicating that both of these subsets may be activated (Fig. 3E). The expression of 4‐1BB was higher in DP compared to CD28^+^CD57^−^, DN, and CD28^−^CD57^+^ subsets (Fig. 3F). Upon T‐cell activation, the expression of co‐inhibitory receptors is induced on T cells 22. To further examine the activation state of the four CD8^+^ T cell subsets, we compared the expression of co‐inhibitory molecules PD‐1, CTLA‐4, and TIGIT between the four subsets (Fig. 3G–I; Supporting Information Fig. 4G–I). Increased frequency of both PD‐1^+^ and CTLA‐4^+^ cells was observed within the DP subset in comparison with CD28^+^CD57^−^, DN, and CD28^−^CD57^+^ cells (Fig. 3G and H). The expression of TIGIT was also higher in DP compared with CD28^+^CD57^−^ and DN cells, but it was not different between DP and CD28^−^CD57^+^ cells (Fig. 3I). HLA‐DR has recently been suggested as a marker for natural human CD8^+^ regulatory T cells 23, 24. Interestingly, the frequency of HLA‐DR^+^ cells was significantly higher within DP cells in comparison to the other CD8^+^ T cell subsets (Fig. 3J; Supporting Information Fig. 4J). When activation molecules and co‐inhibitory receptors were measured within the whole CD8^+^ T cell population, no substantial differences were observed (Supporting Information Fig. 5E–J). In summary, our results indicate that, although both DP and DN CD8^+^ T cells are activated, the responsiveness to T cell cytokines, as well as the expression of co‐inhibitory receptors, is different between the two subsets.

### Proliferation and DNA damage are impaired in both DP and DN CD8^+^ T cells

**Figure 4:**
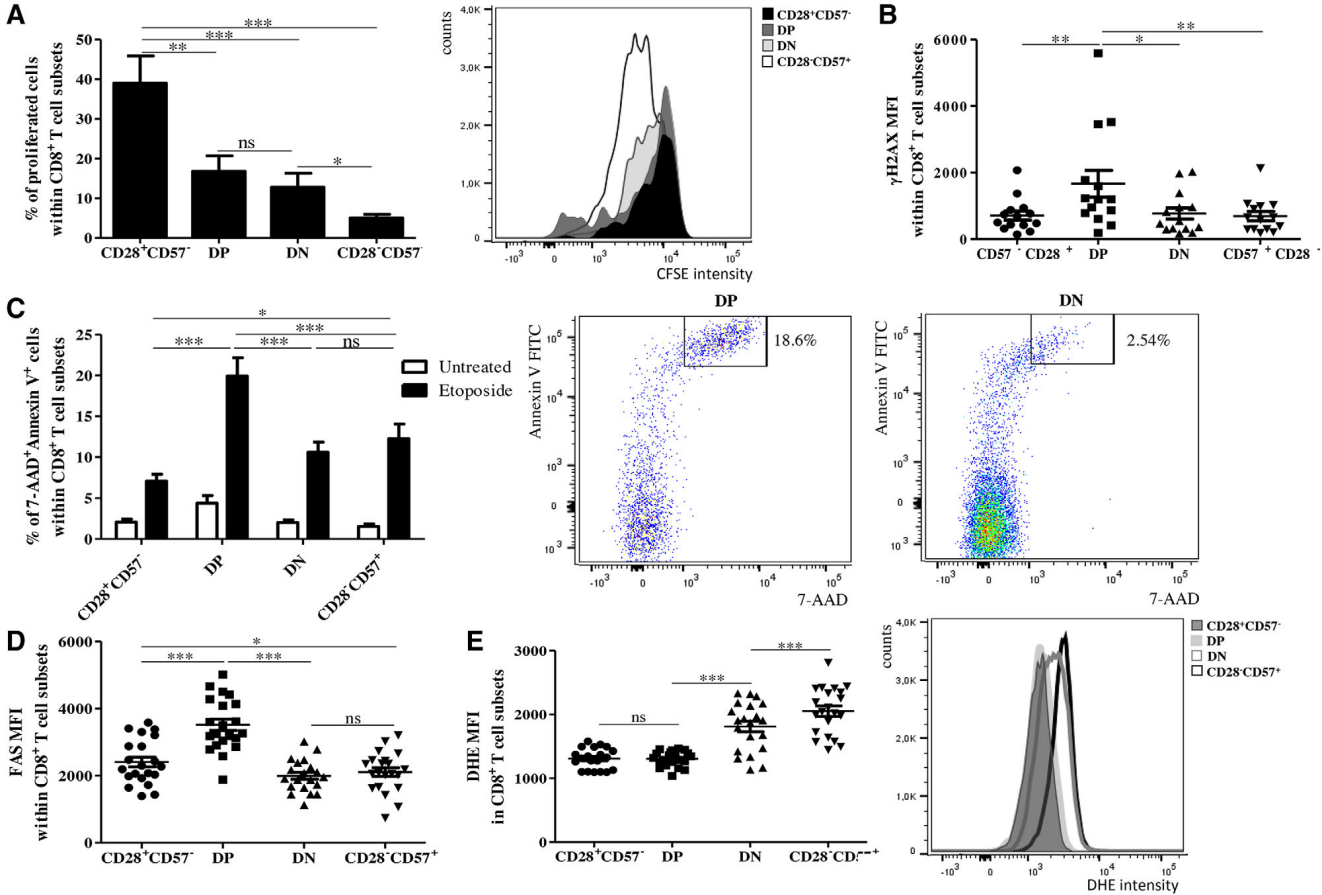
Proliferation, DNA damage, and apoptosis in CD28^+^CD57^−^, DP, DN, and CD28^−^CD57^+^ cells within CCR7^−^CD8^+^ T cells. (A) Percentages of proliferated cells within CD28^+^CD57^−^, DP, DN, and CD28^−^CD57^+^ CCR7^−^CD8^+^ T cells. PBMCs obtained from healthy donors were labeled with CFSE and stimulated with 1 µg/mL α‐CD3 Ab for 4 days. Representative histograms reporting CFSE intensity in each subset is shown. γH2AX MFI (B) and frequency of apoptotic cells (C) in the four subsets within CCR7^−^CD8^+^ T cells. Representative flow cytometry plots showing a 7‐AAD^+^Annexin V^+^ staining in the DP and DN subsets are reported. FAS MFI (D) and ROS levels ( = DHE MFI) (E) in CD28^+^CD57^−^, DP, DN, and CD28^−^CD57^+^ CCR7^−^CD8^+^ T cells. *N* = 5 (A), *N* = 14 (B), *N* = 18 (C), *N* = 21 (D, E). Data are shown as mean ± SEM and pooled from 5 (A), and 14 (B–E) independent experiments. One‐way ANOVA, Bonferroni post hoc test. *p < 0.05; **p < 0.01; ***p < 0.001.

To investigate the proliferative capacity of the DP and DN subsets after antigenic stimulation in comparison with CD28^+^CD57^−^ and CD28^−^CD57^+^ CD8^+^ T cells, we assessed the frequency of proliferated cells within the four cell populations (gated on CCR7^−^ cells) in PBMCs after labeling with CFSE and stimulation with α‐CD3 antibody for 4 days (Fig. 4A). The CD28^+^CD57^−^ subset showed significantly higher proliferation capacity compared to DP and DN cells, which both proliferated more than CD28^−^CD57^+^ cells. No differences were observed between DP and DN cells.

γH2AX levels measured by flow cytometry were higher in DP compared to the other subsets and no differences could be observed between the other subpopulations (Fig. 4B). A representative FACS plot for γH2AX is shown in Supporting Information Figure 6A. To investigate in which subsets DNA damage may preferentially induce apoptosis, PBMCs were treated with etoposide for 2 days and the frequency of apoptotic 7‐AAD^+^Annexin V^+^ cells was measured (Fig. 4C). Increased levels of 7‐AAD^+^Annexin V^+^ cells were observed in DP, while they were lower in DN and CD28^−^CD57^+^ and the lowest in CD28^+^CD57^−^ CCR7^−^CD8^+^ T cells. The overall mRNA expression of genes involved in DNA repair was lower in both DP and DN cells compared to CD28^+^CD57^−^ CD8^+^ T cells (Table 2). While no differences were found between DP and DN cells, a further reduction in the levels of DNA repair genes was seen in CD28^−^CD57^+^ CD8^+^ T cells, in comparison with DP and DN subsets. Interestingly, genes promoting apoptosis were overexpressed in DP when compared to DN, but also to CD28^+^CD57^−^ and CD28^−^CD57^+^ subsets, suggesting that, only in DP cells, impaired DNA repair systems may lead to DNA damage and finally to apoptosis (Table 2). In addition, Fas protein expression was higher in DP compared to the other subsets and the lowest in CD28^−^CD57^+^ CD8^+^ T cells (Fig. 4D; Supporting Information Fig. 6B). Reactive oxygen species (ROS) have been described to support cellular senescence in fibroblasts 25. When we measured ROS levels, oxygen radicals were higher in DN in comparison to CD28^+^CD57^−^ and DP cells, and the highest in CD28^−^CD57^+^ CCR7^−^CD8^+^ T cells (Fig. 4E). The same results were obtained when all experiments (Fig. 4A–E) were performed on the whole CD8^+^ T cell population, without the gating on CCR7^−^ cells (data not shown).

**Table 2:** Genes coding for proteins involved in DNA repair and apoptosis in CD28^+^CD57^−^, DP, DN, and CD28^−^CD57^+^ CD8^+^ T cells

DNA repair genes	CD28^+^ CD57^−^	DP	DN	CD28^−^ CD57^+^	DP versus CD28^+^CD57^+^	DP versus DN	DP versus CD28^−^CD57^+^	DN versus CD28^+^CD57^−^	DN versus CD28^−^CD57^+^
PDE6G	5.67	4.27	5.55	4.41	*	ns	ns	ns	*
LIG1	7.77	6.38	6.77	6.49	*	ns	ns	*	ns
NME4	12.63	11.10	11.64	12.21	**	ns	*	*	ns
POLH	10.85	9.73	10.02	9.87	***	ns	ns	*	ns
IMPDH2	13.53	12.21	12.42	12.33	***	ns	ns	***	ns
POLR1C	9.74	8.93	8.83	8.67	*	ns	ns	*	ns
RALA	10.02	9.40	9.29	8.93	*	ns	*	***	ns
CSTF3	12.47	11.96	11.46	11.37	*	ns	**	***	ns
POLI	9.61	8.55	8.47	8.65	**	ns	ns	*	ns
FANCC	6.96	5.49	6.28	6.83	*	ns	*	ns	ns

Apoptosis genes	CD28^+^ CD57^−^	DP	DN	CD28^−^ CD57^+^	DP versus CD28^+^CD57^+^	DP versus DN	DP versus CD28^−^CD57^+^	DN versus CD28^+^CD57^−^	DN versus CD28^−^CD57^+^
FAS	9.88	11.68	9.95	10.49	***	***	***	ns	ns
TRADD	6.43	7.41	6.55	6.24	*	*	***	ns	ns
TNFRSF10D	7.66	5.86	5.49	4.52	**	**	*	***	*
TNFRSF10A	10.47	10.47	9.70	8.70	ns	ns	***	ns	*
TNFSF10	10.65	10.08	9.37	8.51	ns	ns	*	*	*
DTHD1	6.74	9.89	8.80	8.48	***	***	*	***	ns
RASGEF1A	8.11	11.39	11.31	10.43	***	ns	**	***	*
CD70	5.18	7.52	5.57	6.07	**	*	**	ns	*
PMAIP1	5.03	7.45	6.53	6.39	**	ns	ns	ns	ns

The relative gene expression of each gene measured using microarrays is reported. Within one gene, lower expression is shown in blue, higher expression in red, and intermediate levels are reported in purple. One‐way ANOVA, Bonferroni post hoc test. *p < 0.05; **p < 0.01; ***p < 0.001. *N* = 4 for each subset. Data are combined from one representative experiment.

In summary, our results indicate that proliferation and DNA repair are impaired in both the DP and DN CD8^+^ T cell subsets. While DP cells are highly pro‐apoptotic, apoptosis is inhibited in the CD28^−^ subsets. Specifically, CD28^−^CD57^+^ CD8^+^ T cells show features typical of senescent cells.

### Expression of chemokine receptors is increased in DP in comparison with DN CD8^+^ T cells

**Figure 5:**
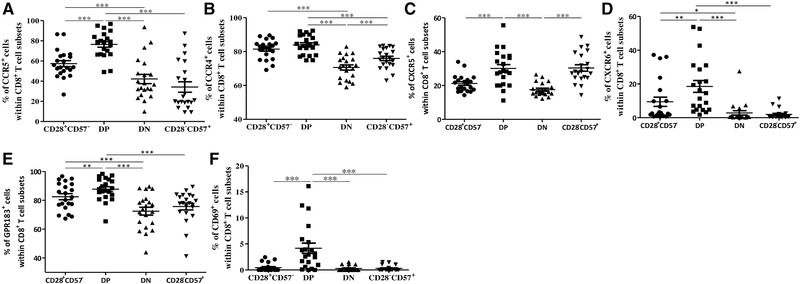
Expression of chemokine receptors and molecules involved in migration in CD28^+^CD57^−^, DP, DN, and CD28^−^CD57^+^ cells within CCR7^−^CD8^+^ T cells. Frequency of CCR5^+^ (A), CCR4^+^ (B), CXCR5^+^ (C), CXCR6^+^ (D), GPR183^+^ (E), and CD69^+^ (F) cells within CD28^+^CD57^−^, DP, DN, and CD28^−^CD57^+^CCR7^−^CD8^+^ T cells within PBMCs obtained from healthy donors measured using flow cytometry. *N* = 21 in each graph. Data are shown as mean ± SEM and pooled from 14 independent experiments. One‐way ANOVA, Bonferroni post hoc test. *p < 0.05; **p < 0.01; ***p < 0.001.

After their activation, CD8^+^ T cells increase the expression of chemokine receptors, which allow them to migrate. The gene expression of selected genes coding for chemokine receptors, chemokines, and molecules involved in cell migration was measured using microarrays is reported in Table 3. Interestingly, the expression of all these molecules was high in the DP cells, suggesting a high migration capacity for this subset. In contrast, expression levels of chemokine receptors were low in CD28^−^CD57^+^ CD8^+^ T cells. Recently activated, but not naïve, CD8^+^ T cells overexpress CCR5, enabling them to respond to CCL3/4 produced by APCs 26. Thus, we investigated the expression of CCR5 on CD28^+^CD57^−^, DP, DN, and CD28^−^CD57^+^ CD8^+^ T cells (Fig. 5A; Table 3; Supporting Information Fig. 6C–H). All the parameters measured by FACS were assessed within CCR7^−^ effector cells. Despite this, no significant differences were observed when the whole CD8^+^ T cell population was considered (data not shown). DP cells showed the highest levels of CCR5 when compared with DN and to the other subsets. At the protein level, DN cells expressed significantly less CCR5 than the CD28^+^CD57^−^ subsets, but the levels were similar when compared to CD28^−^CD57^+^ CD8^+^ T cells (Fig. 5A and Table 3). In addition, the expression of CCR4, mainly associated with migration to non‐lymphoid tissues 27, was increased in CD28^+^CD57^−^ and DP compared with DN and CD28^−^CD57^+^ subsets (Fig. 5B). CXCR5 has been shown to be critical for entering the B cell zones in secondary lymphoid organs 28. When we measured CXCR5 protein expression within the four CD8^+^ T cell subsets, the levels of this chemokine receptors were increased in DP and CD28^−^CD57^+^ compared to CD28^+^CD57^−^ and DN cells (Fig. 5C). In addition, the expression of CXCR6 was increased in DP compared to the other subsets, while it was the lowest within CD28^−^CD57^+^ cells (Fig. 5D and Table 3). GPR183 (EBI2), which has been shown to be important in immune cell migration 29, was high in CD28^+^CD57^−^, the highest in DP cells, but it was significantly reduced in the DN and CD28^−^CD57^+^ subsets (Fig. 5E). CD69 has been indicated as a marker for T cell activation, and recently, as a molecule involved in cell migration important for T cell homing to lymph nodes and to the BM 30, 31. When the expression of CD69 was quantified in CD28^+^CD57^−,^ DP, DN, and CD28^−^CD57^+^ CD8^+^ T cells, it was higher in DP when compared with the other subsets (Fig. 5F). No differences were found between the subsets in the expression of CXCR4 (data not shown).

**Table 3:** Genes coding for chemokine receptors, chemokines, and molecules involved in migration in CD28^+^CD57^−^, DP, DN, and CD28^−^CD57^+^ CD8^+^ T cells

	CD28^+^ CD57^−^	DP	DN	CD28^−^ CD57^+^	DP versus CD28^+^CD57^+^	DP versus DN	DP versus CD28^−^CD57^+^	DN versus CD28^+^CD57^−^	DN versus CD28^−^CD57^+^
CCR5	9.96	11.97	9.96	9.72	*	*	*	ns	ns
CXCR5	7.31	10.63	6.81	6.00	***	***	***	ns	*
CCR4	10.18	11.77	5.45	5.00	ns	***	***	***	ns
CCR7	17.06	14.80	13.41	7.48	***	*	***	***	***
CCRL2	6.51	8.18	8.08	6.92	***	ns	*	***	ns
CXCR3	12.21	13.25	12.93	12.44	ns	ns	*	ns	*
CCR2	9.17	8.83	5.28	5.65	ns	***	***	***	ns
CXCR6	8.67	9.64	8.25	7.61	ns	ns	*	ns	ns
CXCL16	6.16	6.97	5.60	4.77	ns	*	***	ns	ns
GPR183	13.97	14.54	12.22	10.40	ns	***	***	*	*
SEMA4A	6.84	9.22	8.06	7.82	***	*	***	***	ns

The relative gene expression of each gene measured using microarrays is reported. Within one gene, lower expression is shown in blue, higher expression in red, and intermediate levels are reported in purple. One‐way ANOVA, Bonferroni post hoc test. *p < 0.05; ***p < 0.001. *N* = 4 for each subset. Data are combined from one representative experiment.

**Figure 6:**
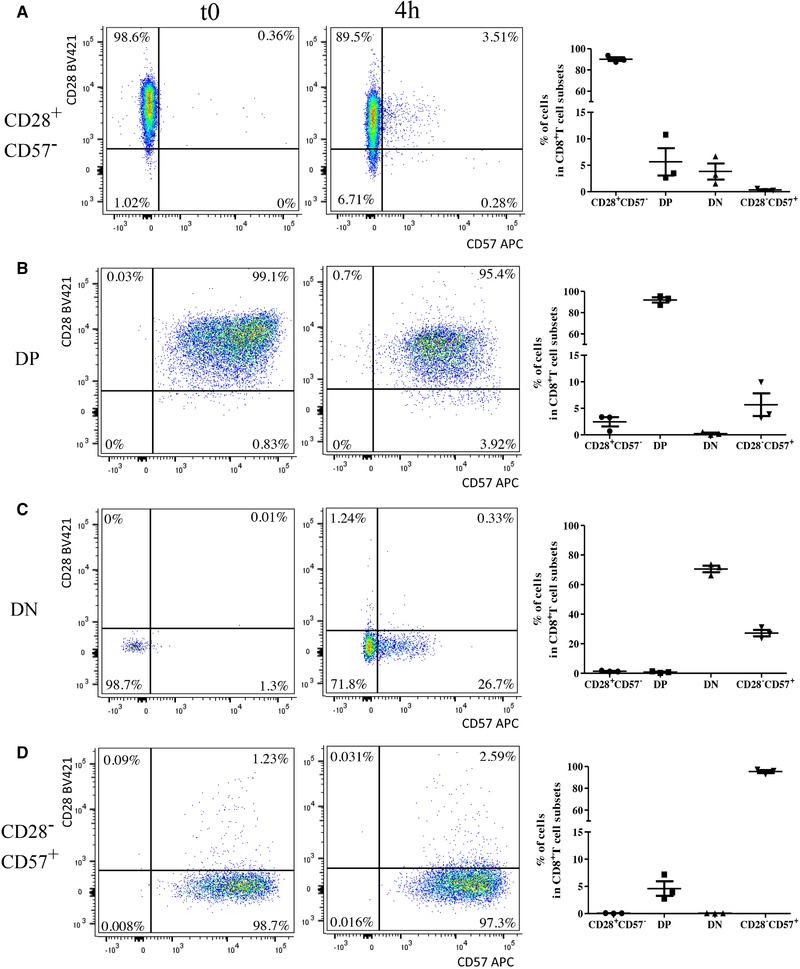
Differentiation potential of sorted CD28^+^CD57^−^, DP, DN, and CD28^−^CD57^+^ CD8^+^ T cells. CD28^+^CD57^−^ (A), DP (B), DN (C), and CD28^−^CD57^+^ (D) CD8^+^ T cells, sorted from PBMCs obtained from healthy donors, were incubated in the presence of 1 µg/mL α‐CD3 and 10 ng/mL IL‐2 for 4 h and stained with anti‐CD3, anti‐CD8, anti‐CD57, and anti‐CD28 Abs. Representative flow cytometry plots at *t*0 and after 4 h are shown. For each condition, frequency of CD28^+^CD57^−^, DP, DN, and CD28^−^CD57^+^ CD8^+^ T cells after 4 h of incubation is shown. Donor *N* = 3. Data are shown as mean ± SEM and pooled from three independent experiments performed separately.

Overall, these results suggest that DP CD8^+^ T cells may show increased migration potential in comparison to CD28^+^CD57^−^, DN, and CD28^−^CD57^+^ CD8^+^ T cells.

### DP and DN cells can both be generated from CD28^+^CD57^−^CD8^+^ T cells

In order to assess how DP and DN cells may be generated and whether these populations are stable or in an “in‐transition” status, FACS sorted CD28^+^CD57^−,^ DP, DN, and CD28^−^CD57^+^ CD8^+^ T cells were cultured for 4 h in the presence of α‐CD3 and IL‐2 (Fig. 6). After the incubation, both DP and DN cells and a small amount of CD28^−^CD57^+^ cells were generated from CD28^+^CD57^−^ CD8^+^ T cells (Fig. 6A). When DP cells were cultured, they partially (5.7 ± 3.7%) lost CD28 from their surface, therefore differentiating into CD28^−^CD57^+^ cells (Fig. 6B). Despite this, DP CD8^+^ T cells were stable in culture. Conversely, the DN subset was very unstable, as high frequency of these cells (27.2 ± 3.8%) expressed CD57 and differentiated into CD28^−^CD57^+^ CD8^+^ T cells (Fig. 6C). Finally, when purified CD28^−^CD57^+^ CD8^+^ T cells were put in culture, no significant changes could be observed (Fig. 6D). Similar results were obtained when the cells were incubated for 12 and 24 h (Supporting Information Table 1). In summary, our findings indicate that, once generated from the CD28^+^CD57^−^ subset, DP CD8^+^ T cells are stable in culture up to 4 h, while high frequency of DN CD8^+^ T cells rapidly differentiate into CD28^−^CD57^+^ cells.

### Frequencies of DP cells increase in the BM and after HBV vaccination

**Figure 7:**
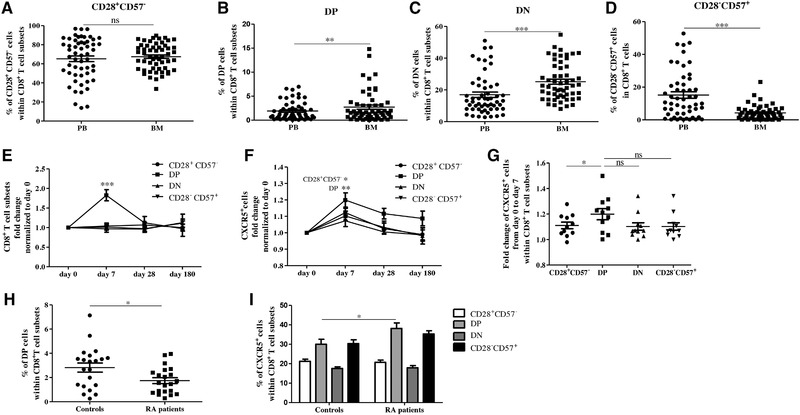
CD28^+^CD57^−^, DP, DN, and CD28^−^CD57^+^ CD8^+^ T cells in the BM, after HBV vaccination and in RA patients. Frequency of CD28^+^CD57^−^ (A), DP (B), DN (C), and CD28^−^CD57^+^ (D) CD8^+^ T cells in paired PBMC and BMMC samples obtained from healthy donors. Data are shown as mean ± SEM and pooled from 55 independent experiments with one sample/experiment. Paired *t*‐test, donor *N* = 55. Fold change of CD28^+^CD57^−^, DP, DN, and CD28^−^CD57^+^ CD8^+^ T cells (E) and CXCR5^+^CD28^+^CD57^−^, CXCR5^+^DP, CXCR5^+^DN, and CXCR5^+^CD28^−^CD57^+^ CD8^+^ T cells (F) on day 0, day 7, day 28, and day 180 after HBV vaccination. The frequency of each population on day 0 is considered as 1. One‐way ANOVA, Bonferroni post hoc test. *N* = 11. (G) Fold change of CXCR5^+^CD28^+^CD57^−^, CXCR5^+^ DN, CXCR5^+^ DP, CXCR5^+^CD28^−^CD57^+^ CD8^+^ T cells from day 0 to day 7. Data are shown as mean ± SEM and obtained from a single representative experiment performed with all donors together. *p < 0.05; **p < 0.01; ***p < 0.001. (H) Frequency of DP CD8^+^ T cells in RA patients and healthy (age‐matched) controls. Paired *t*‐test, *N* = 21 in each group. *p = 0.02. (I) Percentages of CXCR5^+^ cells within CD28^+^CD57^−^, DP, DN, and CD28^−^CD57^+^ CD8^+^ T cells in RA patients and healthy controls. One‐way ANOVA, donor *N* = 21. *p = 0.03. Data are shown as mean ± SEM and obtained from 21 independent experiments with one sample per experiment.

The importance of the BM in the long‐term maintenance of antigen experienced T cells has been extensively documented 4, 5, 6. In order to reach their final position in the survival niches, effector/memory T cells must actively migrate into and within the BM matrix. To assess whether subsets with improved migratory potential, such as DP cells, may be enriched in the BM, we compared the frequency of CD28^+^CD57^−^, DP, DN, and CD28^−^ CD57^+^ populations within CD8^+^ T cells, in paired PBMC and BMMC samples (Fig. 7A–D). While no differences were found for CD28^+^CD57^−^ CD8^+^ T cells, accumulation of DP and DN cells and a reduced frequency of CD28^−^CD57^+^ cells were observed in BMMCs compared with PBMCs. Thus, the increased migration capacity of this subset may support the accumulation of DP cells in the BM. The impaired migration of CD28^−^CD57^+^ CD8^+^ T cells may exclude this population from the BM, promoting an enrichment in the DN subset.

To assess how CD28^+^CD57^−^, DP, DN, and CD28^−^CD57^+^ CD8^+^ T cells may react during an active immunization in vivo, human hepatitis B (HBV) vaccination was used as a model (Fig. 7E–G). The frequency of the four subsets was measured on day 0, day 7, day 28, and day 180 after a booster dose of HBV vaccine (Fig. 7E). While the frequency of CD28^+^CD57^−^, DN, and CD28^−^CD57^+^ CD8^+^ T cells did not change following the vaccination, the levels of DP cells increased on day 7. The frequency of DP cells was back at the pre‐vaccination level by day 28 and day 180. In addition, the expression of CXCR5 increased 7 days after vaccination in CD28^+^CD57^−^ and DP, but not in DN and CD28^−^CD57^+^CD8^+^ T cells (Fig. 7F). While in the CD28^+^CD57^−^ subset the frequency of CXCR5^+^ cells on day 180 was similar to day 0, CXCR5^+^ DP cells were stable up to day 180. In addition, CXCR5 upregulation on day 7 was significantly higher in DP compared to CD28^+^CD57^−^ cells (Fig. 7G). No differences were observed in the expression of CCR5, CCR4, and CXCR6 in any of the subsets after vaccination (data not shown).

In order to investigate whether DP CD8^+^ T cells may change in clinical settings, frequency of this subset was measured in the PB of rheumatoid arthritis (RA) patients and healthy age‐matched controls (Fig. 7H and I). Decreased levels of DP cells could be observed with RA in comparison to the healthy group (Fig. 7H). In addition, the frequency of CXCR5^+^ cells increased in the DP subset of RA patients in relationship to the controls. No significant differences were observed for the other subpopulations. In summary, the levels of DP and CXCR5^+^ DP CD8^+^ T cells change both 7 days following HBV booster vaccination and in patients with RA. This suggests that DP CD8^+^ T cells may play a specific role during immune responses.

## Discussion

With our work, the phenotype of CD28^+^CD57^+^(DP) CD8^+^ T cells in the periphery was characterized for the first time, comparing it with CD28^−^CD57^−^(DN), CD28^+^CD57^−^, and CD28^−^ CD57^+^CD8^+^ T cells. CD57 per‐cell expression was higher in CD28^−^CD57^+^ CD8^+^ T cells in comparison to DP cells, in which only CD57^dim^ cells were observed (Fig. 1A and B). This raises the possibility that a low expression of CD57 may be required during the early activation phase of CD8^+^ T cells, which may then increase in late‐differentiated cells.

As CMV has been shown to drive T cells to terminal differentiation 32, 33, the frequency of CD28^+^CD57^−^, DP, DN, and CD28^−^CD57^+^CD8^+^ T cells in the PB of CMV seropositive and seronegative persons of different age was compared. Similar to previous results, CD28^+^CD57^−^ cells decreased and CD28^−^CD57^+^ cells increased during aging in CMV^−^ and CMV^+^ persons 18. We showed that the overall frequency of DP cells within CD8^+^ T cells did not change with age, but, when the samples were divided into CMV^−^ and CMV^+^ persons, DP CD8^+^ T cells increased significantly with age in CMV^−^ donors (Fig. 1C). Thus, as the phenotype of DP cells is similar to CD28^+^CD57^−^ cells, showing a particularly high frequency within the naïve, CM, and EM CD8^+^ T cell subsets, we can hypothesize that increased numbers of DP cells in old age may be beneficial for immune responses. CMV supports the accumulation of more differentiated CD8^+^ T cell subsets, such as DN and CD28^−^CD57^+^ CD8^+^ T cells, which are mostly EM and TEMRA. Indeed, with age, a positive trend was found for DN CD8^+^ T cells in CMV^+^ persons. As DN CD8^+^ T cells are mainly CCR7^−^, this cell subset is excluded from lymph nodes, similarly to CD28^−^CD57^+^ CD8^+^ T cells, and may preferentially home to non‐lymphoid tissues.

To better compare DP and DN CD8^+^ T cells with CD28^+^CD57^−^ and CD28^−^CD57^+^ CD8^+^ T cells, we first measured the expression of markers of CD8^+^ T cell differentiation in the four subpopulations using microarrays. We clearly described the DP and DN subsets as more differentiated than CD28^+^CD57^−^ cells; however, they are not terminally differentiated like CD28^−^CD57^+^ CD8^+^ T cells. DP cells show a less pronounced T cell effector state in comparison to DN cells. CX3CR1, a reliable marker for CD8^+^ T cell differentiation 19, was almost undetectable in CD28^+^CD57^−^ CD8^+^ T cells, expressed by approximately half of DP and DN cells and by around 80% of CD28^−^CD57^+^ CD8^+^ T cells. Although CX3CR1 expression was similar between the DP and DN subsets, DN expressed higher levels of T cell effector and senescence‐associated molecules, and NK receptors known to be upregulated with CD8^+^ T cell differentiation 21.

In order to exclude that the observed differences were not influenced by the heterogeneity within the four subsets, the expression of all the parameters measured at the protein level was assessed in effectors (CCR7^−^) and in the whole (CCR7^+/−^) CD8^+^ T cell populations. Interestingly, very similar results were obtained, indicating that CD28^+^CD57^−^, DP, DN, and CD28^−^CD57^+^ cells represent distinct populations, each with a homogeneous phenotype.

High levels of the pro‐inflammatory cytokine IFN‐γ in CD28^−^ and CD57^+^ CD8^+^ T cells have previously been documented 34, 35, 36. We now show that, while DP cells, similarly to CD28^−^CD57^+^ CD8^+^ T cells, produce quite high levels of TNF and IFN‐γ, reduced levels of both cytokines can be observed in the DN subset. Intermediate levels of both cytokines were found in CD28^+^CD57^−^ CD8^+^ T cells. Thus, we can now suggest that the downregulation of CD28 is not sufficient to support the overproduction of pro‐inflammatory cytokines by T cells, which is achieved only after the expression of terminal differentiation marker CD57. ROS levels were low in CD28^+^CD57^−^ and DP CD8^+^ T cells, intermediate in DN and very high in CD28^−^CD57^+^ CD8^+^ T cells. IFN‐γ 37 and ROS 25 are known to drive cellular senescence, leading to the inhibition of cell proliferation. Indeed, low proliferation and reduced expression of genes coding for molecules involved in the cell cycle (data not shown) were found in CD28^−^CD57^+^ CD8^+^ T cells. Reduced proliferation was also observed in DP and DN cells when compared with CD28^+^CD57^−^ CD8^+^ T cells. Despite this, both cell populations were able to proliferate better than CD28^−^CD57^+^ CD8^+^ T cells, suggesting that these two subsets are not terminally differentiated. While DNA repair genes were highly expressed in the CD28^+^CD57^−^ subpopulation, low in DP and DN cells and very low in CD28^−^CD57^+^ CD8^+^ T cells, apoptosis genes were overexpressed in DP, but not in DN and CD28^−^CD57^+^ CD8^+^ T cells. In addition, the sensitivity to DNA damage was also increased in DP cells. Thus, in the DP subset, activation may cause DNA damage, which may then lead to apoptosis as a consequence. Similarly to DP cells, DNA repair was also impaired in DN CD8^+^ T cells, but the tendency to undergo apoptosis was reduced compared to the DP subset. Indeed, apoptosis is known to be reduced in CD28^−^ compared with CD28^+^CD8^+^ T cells 38. High ROS levels in DN and, even more, in CD28^−^CD57^+^ CD8^+^ T cells, may support their highly differentiated/senescent phenotype. This is in accordance to the report of Callender et al. 39, wherein the expression of SASP genes was found to be higher in CD8^+^ TEMRA cells, a cell subset enriched in both DN and CD28^−^CD57^+^ CD8^+^ T cells.

In addition to the differences in their differentiation state, DP and DN CD8^+^ T cells are also different regarding their responsiveness to T cell cytokines. The expression of cytokine receptors IL‐7Rα, IL‐6Rα, and CD25 was lower in DN compared to DP, while CD122, a marker for IL‐15 responsiveness, was higher in DN in comparison to the other subsets. Thus, although the responsiveness to IL‐7, IL‐6, and IL‐2 was quite low, DN cells may still respond well to IL‐15. The expression of IL‐15 increases in the BM in the elderly 11, 33. We now observed increased levels of DN CD8^+^ T cells in the BM in old age, suggesting that IL‐15 may contribute to the survival of this subset. CD25 was very low in both DN and CD28^−^CD57^+^ CD8^+^ T cells, therefore, IL‐2 does not seem to play a role in the maintenance of these two cell subsets of highly differentiated cells.

High levels of CCR5, CCR4, and GPR183 on DP CD8^+^ T cells indicate that this population may show increased migration potential to lymphoid and non‐lymphoid organs in comparison to the other three CD8^+^ T cell subsets. In addition, DP cells overexpressed CD69, which is used as a marker for T cell activation, but is also involved in cell migration 30, 31. CD69 allows T cells to enter into the BM, and plays an important role as a retention marker, allowing effector/memory T cells to be maintained there for long periods of time 31. In accordance with this, we found that DP cells accumulate in the BM, while CD28^−^CD57^+^ CD8^+^ T cells, which expressed lower levels of the chemokine receptors CCR5, CCR4, and CD69, were mainly excluded and enriched in the PB. In addition, the frequency of DN cells was higher in the BM compared to the PB. We therefore hypothesize that senescent‐like CD8^+^ T cells may not be able to reach T cell survival niches in the BM, most likely because of their impaired expression of chemokine receptors.

Interestingly, DP CD8^+^ T cells showed features of activated cells, with increased expression of co‐stimulatory molecules CD160 and 4‐1BB. In addition, the frequency of co‐inhibitory receptors PD‐1, CTLA‐4, and TIGIT, known to be overexpressed after T cell activation, was higher in DP in comparison to CD28^+^CD57^−^, DN, and CD28^−^CD57^+^ CD8^+^ T cells. A population of HLA‐DR^+^ CD8^+^ regulatory T cells expressing high levels of CTLA‐4 and showing suppressive activity has been described 22, 23. In our study, we observed that the frequency of HLA‐DR^+^ cells was high within DP CD8^+^ T cells, while it was lower in the other three subsets. In addition, DP CD8^+^ T cells were shown to express high levels of IL‐10, a cytokine typically expressed by regulatory T cells. T cells require chemokine receptor CXCR5 to enter the B cell zones in secondary lymphoid organs 28. Indeed, it has been reported that early CD4^+^ T follicular helper (T_FH_) cells require CXCR5 to migrate to the border of B cell follicles and to start T_FH_ differentiation 40. In parallel, a population of regulatory, T_FH_‐like CD8^+^ T cells expressing CXCR5 with both B cell helper capacities and partial cytotoxic functions has been detected in B‐cell follicles 41. This subset of CXCR5^+^ CD8^+^ T cells is characterized by high levels of ICOS, a co‐stimulatory molecule belonging to the CD28 family, increased HLA‐DR and PD‐1 expression 42. We now showed that CD28^+^CD57^+^ CD8^+^ T cells are activated cells expressing high levels of CXCR5, HLA‐DR, CTLA‐4, PD‐1, and IL‐10, and therefore, they may display the phenotype of regulatory cells. Although CD28^−^CD57^+^ CD8^+^ T cells express high levels of CXCR5, this subset is excluded from secondary lymphoid organs as it lacks the homing marker CCR7.

We next assessed whether DP CD8^+^ T cells may have any relevance in vivo, using human HBV vaccination as a model. Interestingly, the numbers of DP cells increased 7 days after the HBV booster, and, in parallel, CXCR5 in DP cells was overexpressed in comparison to day 0. This allowed us to speculate that this population of CXCR5^hi^CD28^+^CD57^+^ CD8^+^ T cells may expand after HBV vaccination, and home to lymph nodes, in which they may be needed to provide B cell help. This concept is supported in recent reports, in which the importance of CXCR5^+^ CD8^+^ T cells in offering B cell help in HBV antiviral immune responses has been documented 43. DP cells may also migrate to non‐lymphoid tissues such as the BM, in which they can be maintained, ready to respond in case of a secondary immune response. Whether the BM environment may prevent apoptosis in DP CD8^+^ T cells in order to support the survival of this subset is currently unknown.

Decreased frequency of DP and increased levels of CXCR5^+^ DP CD8^+^ T cells were additionally observed in the PB of RA patients in comparison with healthy controls. This raises the possibility that, during immune responses, the presence of a certain amount of DP CD8^+^ T cells may be required. In addition, in pathological conditions such as with RA, the generation of DP cells may be impaired.

An intriguing question is how DP and DN CD8^+^ T cells may be generated and whether the CD28^+^CD57^−^, DP, DN, and CD28^−^CD57^+^ subpopulations are stable or can differentiate into other subsets over time (Fig. 6). With our in vitro experiment using sorted cells, we observed that CD28^+^CD57^−^ cells may generate both the DP and DN subsets. While DP cells, at least for 4 h, were quite stable regarding the expression of CD28 and CD57, high frequency of DN cells differentiated into CD28^−^CD57^+^ CD8^+^ T cells. Thus, our data suggest that DN cells may represent an “in transition” subset, from a stage of naïve‐memory (CD28^+^CD57^−^) to another one of terminal differentiation (CD28^−^CD57^+^). Despite this, DN cells display a phenotype that is different from the other three CD8^+^ T cell subsets. Whether other T cell cytokines may preferentially support the differentiation of one subset into another one is currently under investigation.

We therefore suggest that, after the contact with antigens, CD28^+^CD57^−^ CD8^+^ T cells may express a small amount of CD57 and differentiate into a population of activated CD28^+^CD57^+^ (DP) cells, which, after migrating to secondary lymphoid organs, may provide B cell help. As this population is highly apoptotic, it may rapidly die after performing its task. Alternatively, CD28^+^CD57^−^ CD8^+^ T cells may either be activated, while maintaining CD28 on their surface, or downregulate CD28 and differentiate into CD28^−^CD57^−^ (DN) cells. Cells that do not lose CD28 after activation may then differentiate into “healthy” memory cells, migrate to the BM, and be maintained there. While CD28^+^CD57^−^ CD8^+^ T cells represent a heterogeneous population, DN cells include more differentiated cells, which are early‐senescent, but still display some effector and cytotoxic functions. Thus, DN CD8^+^ T cells may represent a population of functional CD8^+^ T cells, which may invest most of their energy in fighting ongoing infections rather than in proliferating or differentiating into memory cells. Indeed, DN CD8^+^ T cells may hardly be activated after exposure with antigens. Finally, at least some DN cells may rapidly express CD57, acquiring the phenotype of highly differentiated, senescent‐like CD8^+^ T cells, with high ROS levels, impaired proliferation, and overexpression of pro‐inflammatory and T cell cytotoxic molecules. While intermediate expression of effector molecules is required for CD8^+^ T cell functions, an overproduction may be detrimental. Indeed, a negative correlation of diphtheria‐specific antibody concentrations in the plasma with the frequency of CD28^−^CD57^+^ CD8^+^ T cells and IFN‐γ^+^CD28^−^CD57^+^ CD8^+^ T cells was recently observed by our group 18. In addition, a small amount of DP cells may lose CD28 from the surface and differentiate into CD28^−^CD57^+^ cells. Whether CD28^+^CD57^−^ CD8^+^ T cells may directly differentiate into CD28^−^CD57^+^ cells is unknown.

In summary, with our work, four distinct CD8^+^ T cell subsets were characterized using the markers CD28 and CD57: non‐activated/early‐activated CD28^+^CD57^−^, activated CD28^+^CD57^+^, activated/early‐senescent CD28^−^CD57^−^, and terminally differentiated‐senescent‐like CD28^−^CD57^+^ CD8^+^ T cells. These markers may be useful for the general characterization of CD8^+^ T cells before performing detailed analysis, thus being useful as potential biomarkers for diseases. Strategies to counteract the accumulation of highly differentiated T cells and to boost the generation of memory CD28^+^CD57^−^ and activated CD28^+^CD57^+^ CD8^+^ T cells are currently under investigation.

## Materials and methods

### Blood donors and isolation of PBMCs

Fresh blood samples were obtained from 119 healthy donors (61 females, 58 males, age range 27–87 years, mean age 58 ± 14.2 years). The number of donors used for each individual experiment is indicated in the figure legends. Purification of PBMCs from heparinized blood was performed by density gradient centrifugation (Ficoll‐Hypaque). Serum CMV antibody (IgG) titers were determined by ELISA (Virion Serion), according to the specifications given by the manufacturer. Forty‐eight donors were CMV seronegative and 71 CMV seropositive. PBMCs from donors before and after vaccination against Hepatitis B were frozen samples remaining from a previously published study 44. For the current study, samples from 11 donors were used (age range 23–74, mean age 51 ± 9.7 years), which had received a primary series (three doses) of vaccination against Hepatitis B more than 10 years earlier and a booster dose in the context of the abovementioned study. Fresh PBMCs from rheumatoid arthritis patients were collected from 21 donors (8 males, 13 females, age range 35–78 years, mean age 59 ± 11.3 years) and matched with PBMCs obtained from a group of 21 healthy donors (10 males, 11 females, age range 30–84 years, mean age 54 ± 10.8 years).

### Human BM samples collection and preparation

Paired BM and blood samples were obtained from 55 systemically healthy individuals (20 females, 25 males, age range 36–89 years, mean age 64 ± 12.1 years) who did not receive immunomodulatory drugs or suffer from diseases known to influence the immune system, including autoimmune diseases and cancer. Bone from the femur shaft was harvested during hip replacement surgery, and a biopsy of *substantia spongiosa osseum*, which would otherwise have been discarded, was used to isolate BMMCs. BM biopsies were fragmented, washed once with complete RPMI medium (RPMI 1640 supplemented with 10% FCS, 100 U/mL penicillin, and 100 µg/mL streptomycin; Invitrogen), and treated with purified collagenase (CLSPA, Worthington Biochemical; 20 U/mL in complete RPMI medium) for 1 h at 37°C. BM biopsies were then centrifuged and BMMCs were purified by density gradient centrifugation. Fresh BMMCs and PBMCs were used for the experiments.

### Cell sorting

CD8^+^ T cells were isolated from fresh PBMCs by magnetic cell sorting using the MACS human CD8^+^ T cell isolation kit (Miltenyi Biotech) following the manufacturer's protocol. Purified CD8^+^ T cells were then stained with anti‐CD8 PeVio770, anti‐CD3 APC‐Vio770, anti‐CD57 APC, and anti‐CD28 BV421 antibodies. CD28^+^CD57^−^, CD28^+^CD57^+^, CD28^−^CD57^−^, and CD28^−^CD57^+^ CD8^+^ T cells were sorted with a FACSAria II flow cytometer (BD Biosciences). Sorted cells were collected in complete RPMI 1640 medium and washed once prior to further studies. The purity of each subset was >99% as determined by flow cytometry. Antibodies with non‐activating clones were used for the sorting. Before performing the experiments, cells were rested for 1 h at 37°C in complete RPMI medium. All of the subpopulations showed similar recovery capacity.

### Isolation of RNA

RNA was isolated from sorted cells using the RNeasy Plus mini kit (Qiagen). After the isolation, RNA was quantified and quality was assessed using a Nanodrop (Thermo Scientific). Only RNA samples with a ratio A_260_/A_280_ >2 and a concentration >10 ng/µL were used. CD28^+^CD57^−^, CD28^+^CD57^+^, CD28^−^CD57^−^, and CD28^−^CD57^+^ CD8^+^ T cells sorted from four donors were used for the microarray analysis.

### Affymetrix microarray analysis

In order to compare gene expression of CD28^+^CD57^−^, CD28^+^CD57^+^, CD28^−^CD57^−^, and CD28^−^CD57^+^CD8^+^ T cell subsets, Affymetrix Clariom S microarray chip analysis was used. The Affymetrix Clariom S chip measures gene‐level expression from >20 000 well‐annotated genes and >337 000 transcripts, and includes over 205 800 probes. Microarray analysis was performed by Eurofins Genomics, Denmark. Before the analysis, samples for microarrays were amplified using the Affymetrix GeneChip Pico kit (Thermo Fisher), which allows a minimum RNA amount of 3 ng/sample. For each sample, 5 ng of RNA was sent in 3 µL of RNAse free water to perform the analysis.

To obtain functional information about pathways enriched within CD28^+^CD57^−^, CD28^+^CD57^+^, CD28^−^CD57^−^, and CD28^−^CD57^+^CD8^+^ T cell subsets, Gene Set Enrichment Analysis (GSEA) (Broad Institute, http://www.broadinstitute.org/gsea/index.jsp) was performed, as previously described 45.

### Cell culture and flow cytometric analysis

All the flow cytometry experiments were performed in accordance to the “Guidelines for the use of flow cytometry and cell sorting in immunological studies” 46. Immunofluorescence surface staining was performed by adding a panel of directly conjugated Abs to freshly prepared PBMCs and BMMCs. Dead cells were excluded from the analysis using a fixable viability dye (Zombie Aqua™ Fixable Viability Kit, Biolegend). After surface staining, cells were permeabilized using the Cytofix/Cytoperm kit (BD Pharmingen), and incubated with intracellular Abs. To analyze cytokine expression and DNA damage in CD28^+^CD57^−^, CD28^+^CD57^+^, CD28^−^CD57^−^, and CD28^−^CD57^+^CD8^+^ T cells, PBMCs were previously stimulated for 4 h at 37 °C with 30 ng/mL PMA and 500 ng/mL ionomycin in the presence of 10 mg/mL Brefeldin A (BFA; Sigma–Aldrich). DNA damage was assessed measuring the MFI of γH2AX after intracellular staining with anti‐H2AX pS139‐FITC (REA502) (Miltenyi) Abs.

The following labeled Abs were used: anti‐CD3‐APC‐Vio770 (REA613); anti‐CD8‐PE‐Vio770 (REA734), anti‐CD8‐Viogreen (REA734), anti‐CD57 APC (TB03), anti‐CD57‐FITC (TB03), anti‐CCR7‐FITC (REA546), anti‐CCR7‐Pecy7 (G043H7), anti‐CX3CR1‐PE‐Vio770 (2A9‐1), anti‐IL6Rα‐PeVio770, anti‐CD25‐PE (4E3), anti‐CD122‐FITC (REA167), anti‐4‐1BB‐PE (REA7659), anti‐FAS FITC (DX2), anti‐CCR5 PE‐Vio770 (REA245), anti‐CCR4‐APC (REA279), anti‐CXCR6‐PE (REA458), 7‐AAD purchased from Miltenyi, anti‐PD‐1‐PE (EH12.2H7), anti‐CD28‐BV421 (CD28.2), anti‐CD28‐PE (CD28.2), anti‐CD45RA‐PerCp (HI 100), anti‐CD57‐PerCp (HNK‐1), anti‐IL7Rα‐PerCp (A019D5), anti‐CD160‐PerCp (BY55), anti‐CTLA‐4‐APC (L3D10), anti‐TIGIT‐APC (A15153G), anti‐CXCR5‐PerCp (J252D4), anti‐GPR183‐APC (SA313E4), and anti‐CD69‐PE (FN50) purchased from Biolegend and anti‐IFNγ‐FITC (XMG1.2), anti‐TNF‐FITC (MAb11) BD, anti‐IL‐10‐PE (JES3‐19F1), anti‐HLA‐DR‐PE‐Cy7(G‐46‐6) from BD Biosciences. Labeled cells were measured by an FACSCanto II (BD Biosciences). Data were analyzed using Flowjo v10 software.

### Assessment of differentiation potential

To assess the differentiation potential of CD28^+^CD57^−^, DP, DN, and CD28^−^CD57^+^ CD8^+^ T cells, sorted cells were incubated in the presence of 1 µg/mL α‐CD3 and 10 ng/mL IL‐2 for 4 h. Cells were stained with anti‐CD3, anti‐CD8, anti‐CD57, and anti‐CD28 Abs and measured by FACS.

### Assessment of proliferation

Proliferation was measured after CFSE labeling and stimulation of PBMCs with 1 µg/mL anti‐CD3 Ab (BD Pharmingen) for 4 days. After incubation, cells were stained with anti‐CCR7, anti‐CD8, anti‐CD57, and anti‐CD28 Abs and measured by FACS.

### Assessment of apoptosis

In order to determine apoptosis in in CD28^+^CD57^−^, CD28^+^CD57^+^, CD28^−^CD57^−^, and CD28^−^CD57^+^ CD8^+^ T cells, PBMCs were stimulated for 48 h at 37°C with 30 µg/mL etoposide (Sigma–Aldrich). Apoptotic cells were assessed after surface staining with anti‐CCR7, anti‐CD3, anti‐CD8, anti‐CD28, anti‐CD57 Abs, and 7‐AAD, and incubation with anti‐Annexin V‐FITC (RUO, BD Biosciences). Apoptotic cells were identified as Annexin V^+^ 7‐AAD^+^ cells.

### ROS measurement

ROS levels were measured after incubation of PBMCs with the fluorescent dye dihydroethidium (DHE; Sigma–Aldrich) at a concentration of 1:250 in complete RPMI for 20 min at 37°C. Surface Abs anti‐CCR7, anti‐CD3, anti‐CD8, anti‐CD57, and anti‐CD28 were added at the same time.

### Statistical analysis

Statistical significance was assessed by Spearman correlation analysis, one way ANOVA with Bonferroni post hoc test and paired two‐tailed *t*‐test, as indicated in the figure legends. A *p*‐value < 0.05 was considered significant. Mean ± SEM is shown in each graph. For the GSEA, significant differences were considered as *p* < 0.05 and FDR < 0.25.

### Study approval

The study was approved by the Ethics Committee of the Medical University of Innsbruck. Informed consent for test and publication was given and documented from each patient, in accordance with the Declaration of Helsinki.

## Conflict of interest

The authors declare no commercial or financial conflict of interest.

**Abbreviations**
BMMC: BM mononuclear cells
DHE: dihydroethidium
PB: peripheral blood
PBMC: PB mononuclear cells

## Supporting information

Supporting information
