# Supplementary material for: CD28 and CD57 define four populations with distinct phenotypic properties within human CD8+ T cells
Source: Eur J Immunol. 2019 Dec 4;50(3):363–79. doi: 10.1002/eji.201948362 (PMC7079235; doi:10.1002/eji.201948362)
Supplement: Supplementary file 1 — Supporting information [file EJI-50-363-s001.pdf]

European Journal of Immunology

Supporting Information
for
DOI 10.1002/eji.201948362

Luca Pangrazzi, Jürgen Reidla, José Antonio Carmona Arana, Erin Naismith, Carina Miggitsch, Andreas Meryk, Michael Keller, Adelheid Alma Nora Krause, Franz Leonard Melzer, Klemens Trieb, Michael Schirmer, Beatrix Grubeck-Loebenstein and Birgit Weinberger

CD28 and CD57 define four populations with distinct phenotypic properties within human CD8^{+} T cells

© 2019 The Authors. *European Journal of Immunology* published by WILEY-VCH Verlag GmbH & Co. KGaA, Weinheim.

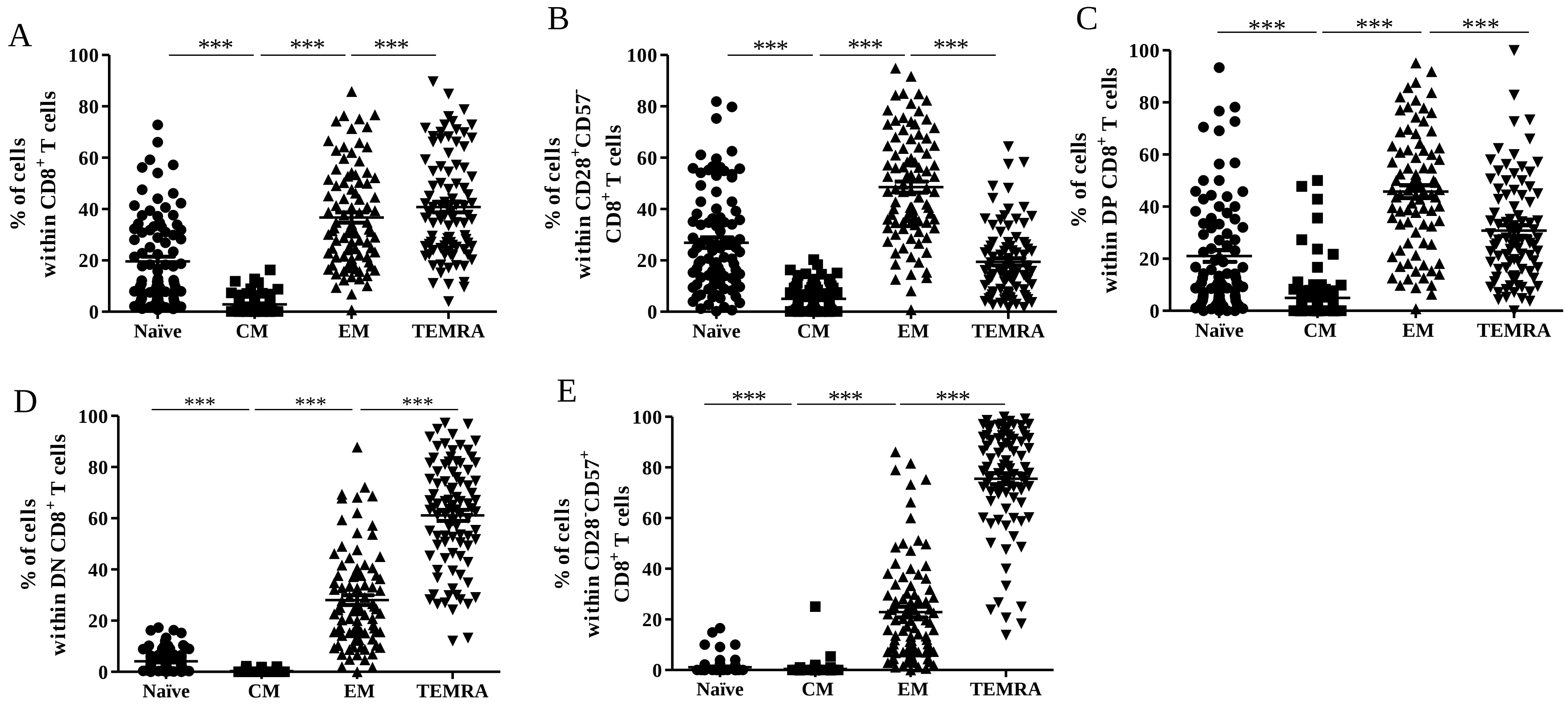

Suppl.Fig. 1. Distribution of naïve, CM, EM and TEMRA**.** Percentages of naïve, CM, EM and TEMRA within CD8$^+$ T cells (A), CD28$^+$CD57$^-$ (B), DP (C), DN (D) and CD28$^-$CD57$^+$ (E) CD8$^+$ T cells. One way ANOVA, Bonferroni *post hoc* test. N=91 in each group. ***$p<0.001$.

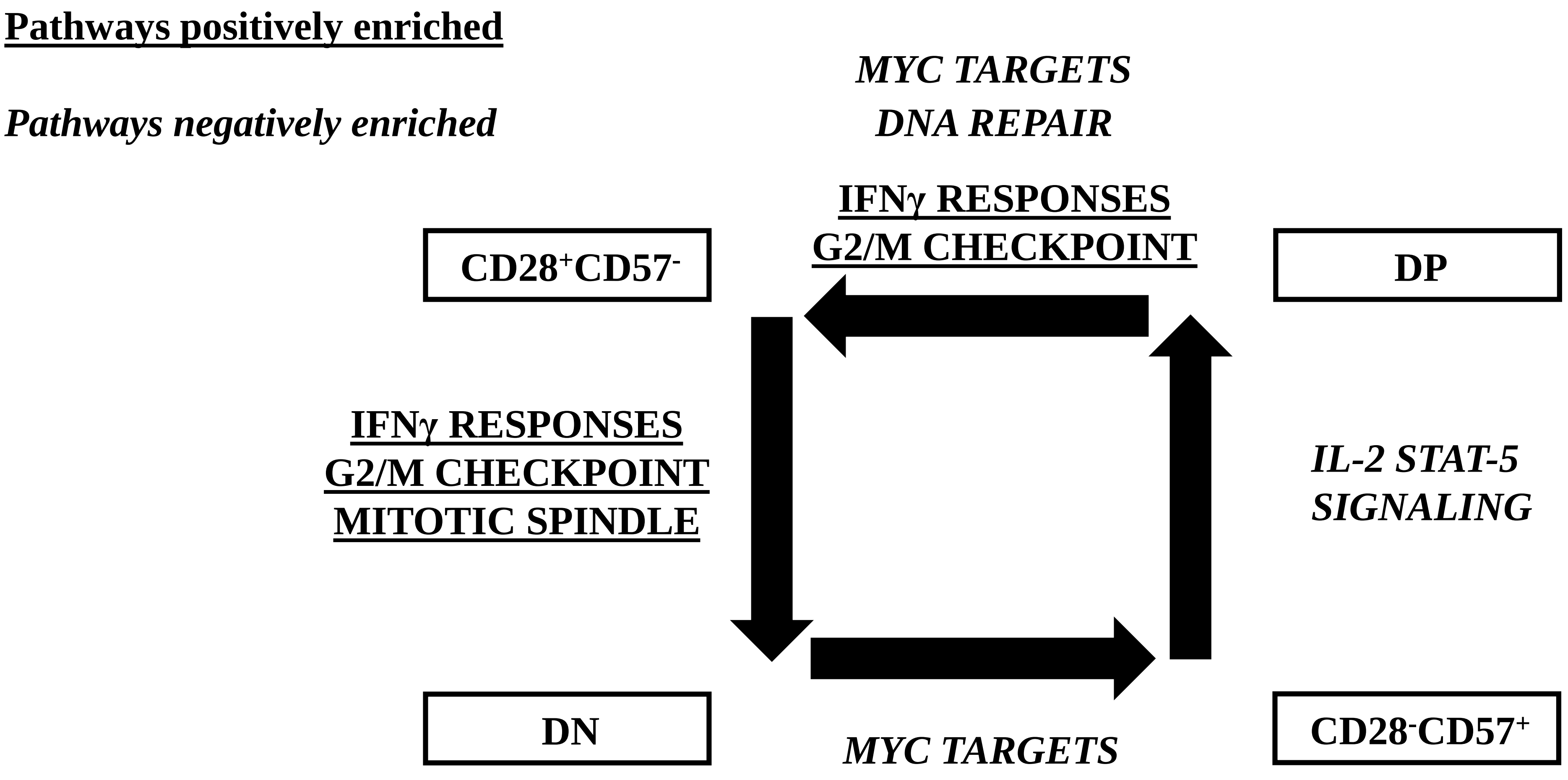

Suppl.Fig. 2. Pathways significantly enriched in the comparisons between $CD28^+CD57^-$, DP, DN and $CD28^-CD57^+CD8^+$ T cells after GSEA analysis. Positively and negative enriched pathways are reported in red and in blue respectively. $p<0.05$, $FDR<0.25$.

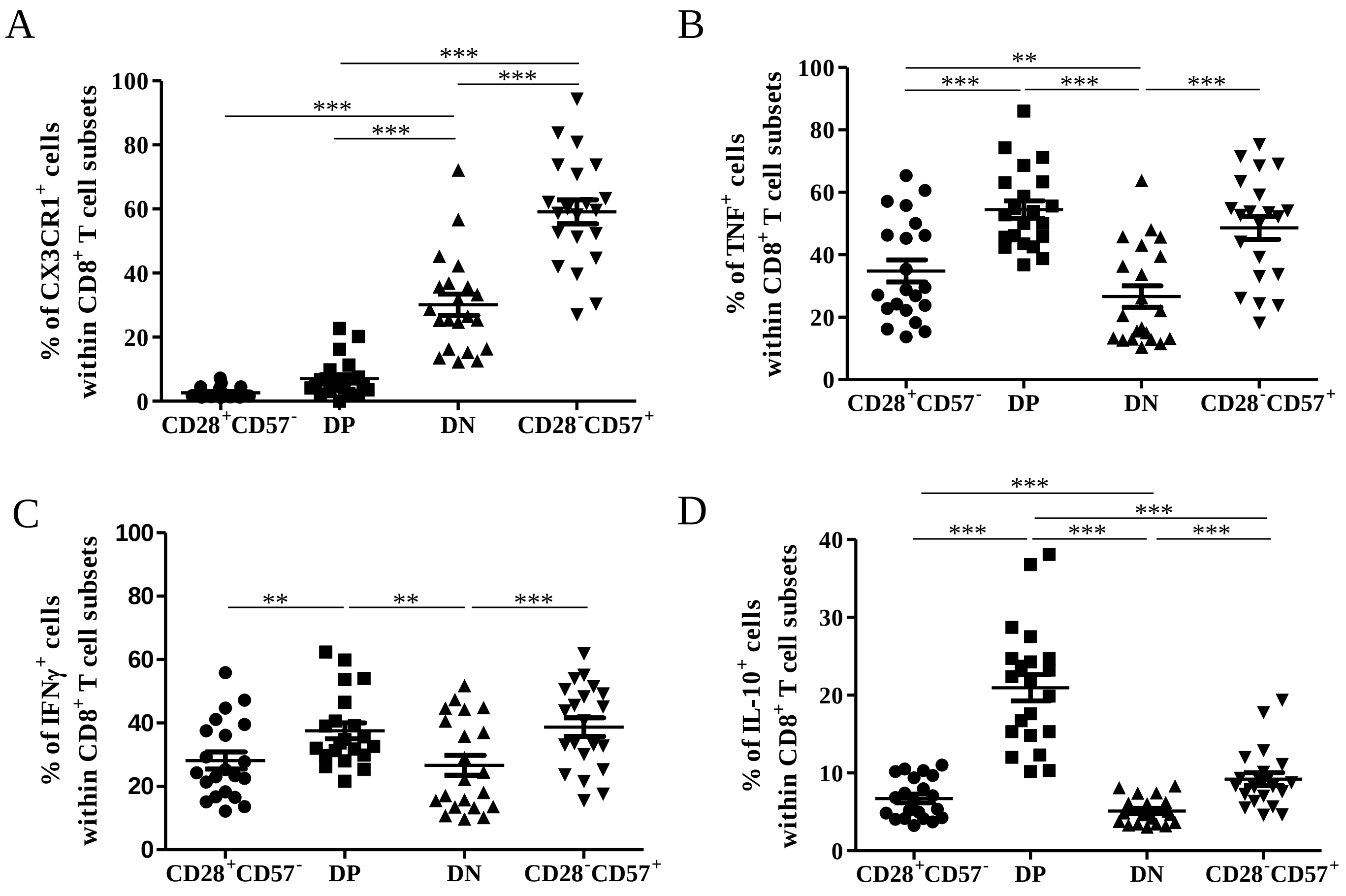

Suppl.Fig. 3. Cytotoxic and effector molecules in CD28$^+$CD57$^-$, DP, DN and CD28$^-$CD57$^+$ cells in the whole CD8$^+$ T cell population. After gating on CD3$^+$CD8$^+$ T cells within lymphocytes, CD28$^+$CD57$^-$, DP, DN and CD28$^-$CD57$^+$ subsets were defined. Frequency of CX3CR1$^+$ (A), TNF$^+$ (B), IFNγ$^+$ (C), IL-10$^+$ (D) cells within CD28$^+$CD57$^-$, DP, DN and CD28$^-$CD57$^+$ CD8$^+$ T cells. N=21 for each graph. Data are shown as mean ± SEM and pooled from 14 independent experiments. One way ANOVA, Bonferroni *post hoc* test. *p<0.05, **p<0.01, ***p<0.001.

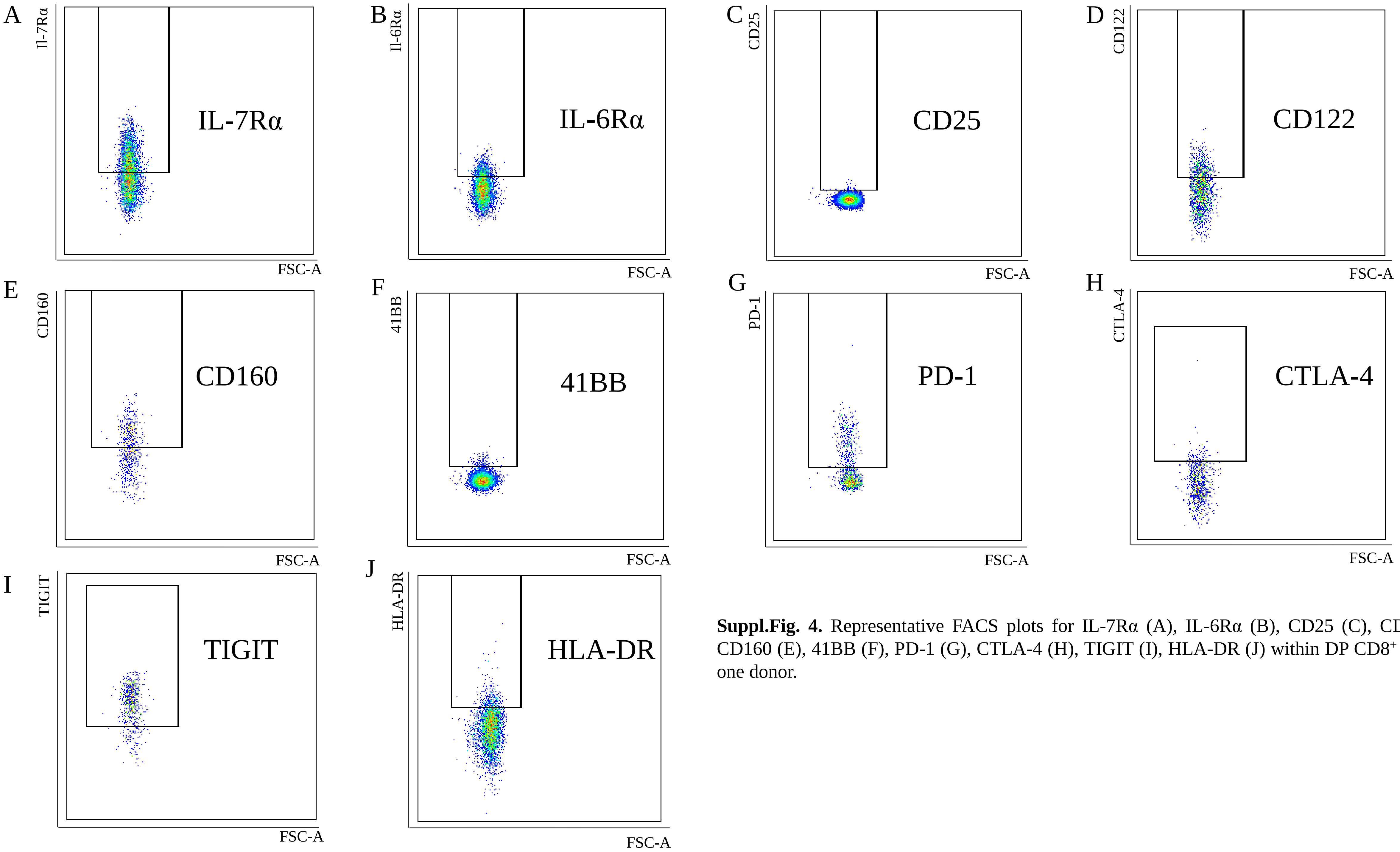

Suppl.Fig. 4. Representative FACS plots for IL-7Rα (A), IL-6Rα (B), CD25 (C), CD122 (D), CD160 (E), 41BB (F), PD-1 (G), CTLA-4 (H), TIGIT (I), HLA-DR (J) within DP $CD8^+$ T cells in one donor.

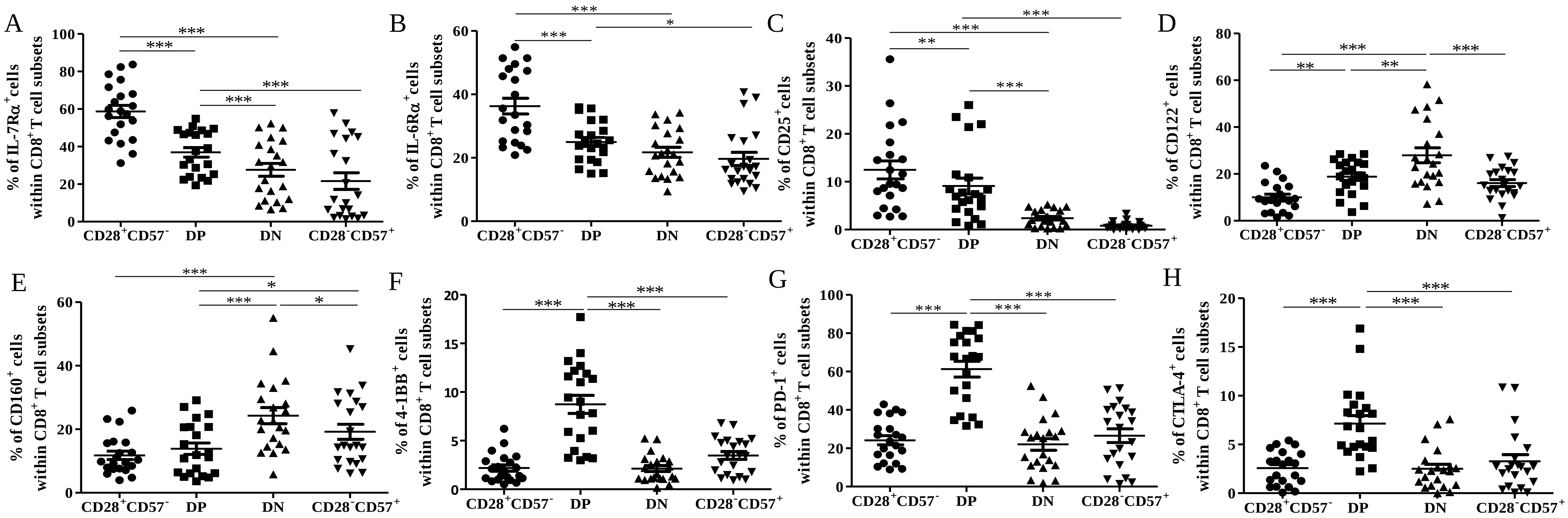

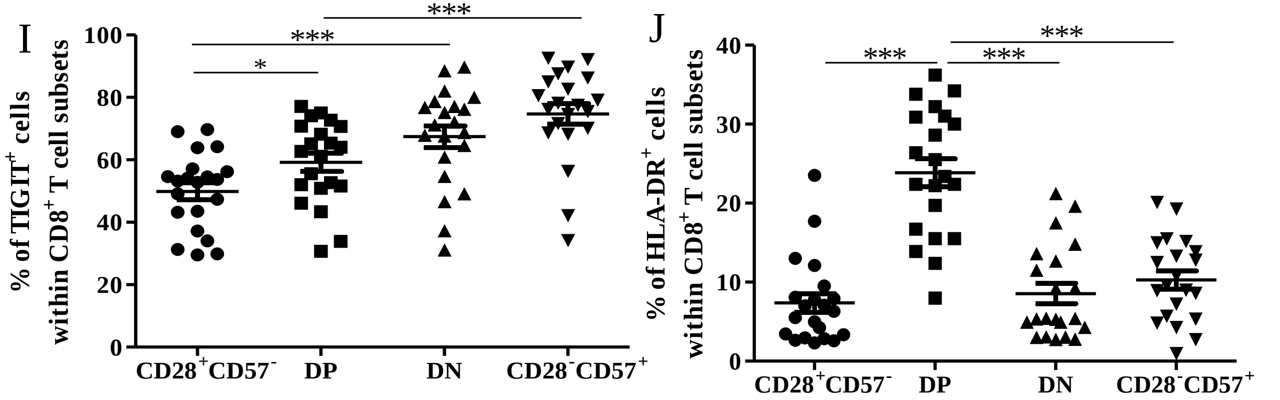

Suppl. Fig. 5. Expression of cytokine receptors, activation and co-inhibitory molecules in CD28$^+$CD57$^-$, DP, DN and CD28$^-$CD57$^+$ cells in the whole CD8$^+$ T cell population. Frequency of IL-7Rα$^+$ (A), IL-6Rα$^+$ (B), CD25$^+$ (C), CD122$^+$ (D), CD160$^+$ (E), 4-1BB$^+$ (F), PD-1$^+$ (G), CTLA-4$^+$ (H), TIGIT$^+$ (I) and HLA-DR$^+$ (J) cells within CD28$^+$CD57$^-$, DP, DN and CD28$^-$CD57$^+$ CD8$^+$ T cells. N=21 for each group. One way ANOVA, Bonferroni post hoc test. *$p<0.05$, **$p<0.01$, ***$p<0.001$. Data are shown as mean ± SEM and pooled from 14 independent experiments.

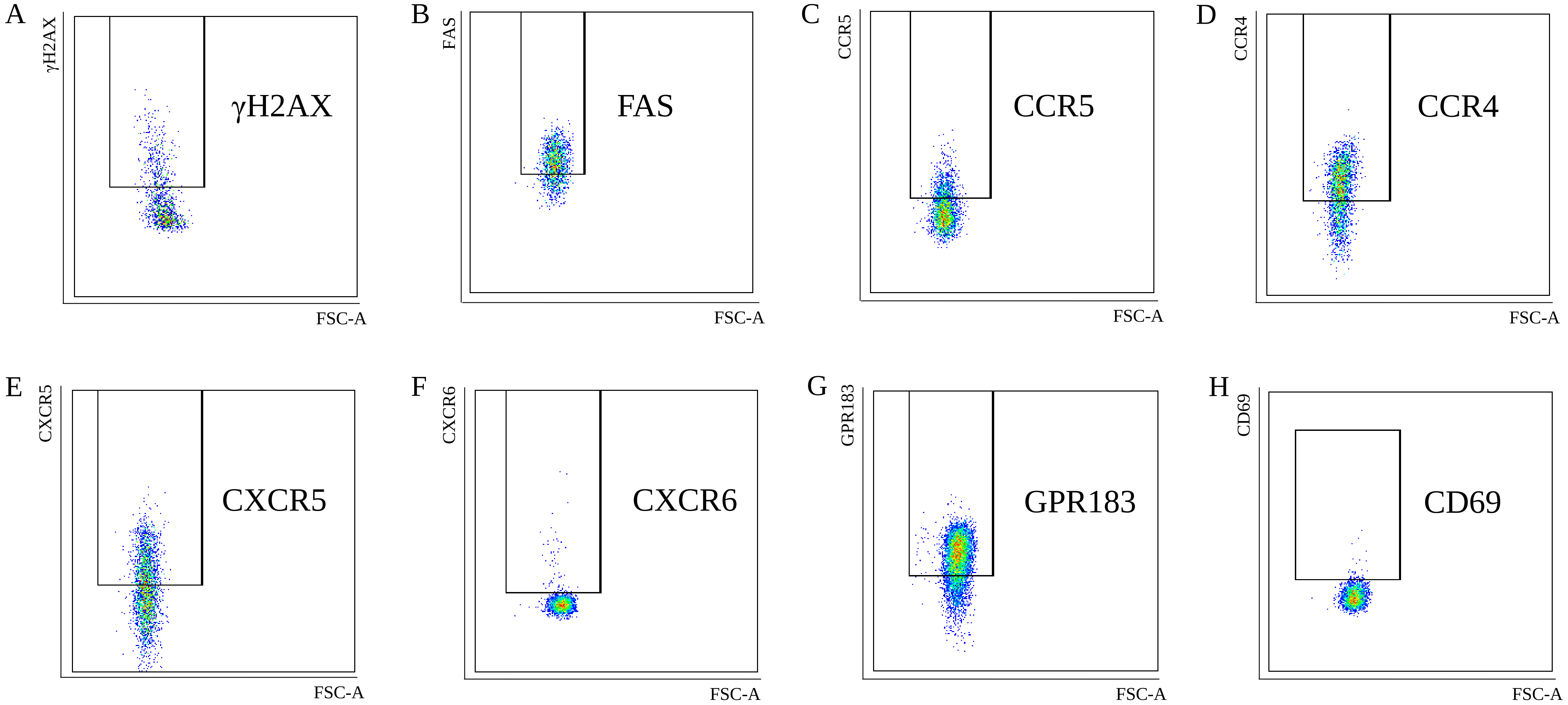

Suppl.Fig. 6. Representative FACS plots for γH2AX (A), FAS (B), CCR5 (C), CCR4 (D), CXCR5 (E), CXCR6 (F), GPR183 (G) and CD69 (H) within DP $CD8^+$ T cells in one donor.

Suppl. Fig. 6

Sorted subsets	Timepoints	Subsets (stained after incubation) CD28$^+$CD57$^-$	DP	DN	CD28$^-$CD57$^+$
**CD28$^+$ CD57$^-$**	4h	90.16 ± 2.5	5.66 ± 3.6	3.83 ± 2.1	0.34 ± 0.2
	12h	87.40 ± 2.4	6.57 ± 3.7	5.52 ± 2.7	0.50 ± 0.2
	24h	86.92 ± 2.6	6.84 ± 3.9	5.57 ± 2.9	0.66 ± 0.1
**DP**	4h	2.45 ± 1.2	91.93 ± 3.5	0.24 ± 0.2	5.69 ± 3.0
	12h	2.56 ± 0.9	90.98 ± 3.2	0.29 ± 0.08	6.15 ± 2.9
	24h	2.89 ± 0.6	90.16 ± 3.1	0.27 ± 0.09	6.66 ± 2.8
**DN**	4h	1.42 ± 0.3	0.84 ± 0.4	70.56 ± 3.1	27.20 ± 3.1
	12h	1.36 ± 0.2	0.75 ± 0.3	66.33 ± 2.8	31.55 ± 2.8
	24h	1.24 ± 0.1	0.77 ± 0.3	63.33 ± 3.7	34.65 ± 3.9
**CD28$^+$ CD57$^-$**	4h	0.05 ± 0.03	4.61 ± 1.9	0.04 ± 0.03	95.33 ± 1.9
	12h	0.03 ± 0.01	3.44 ± 0.7	0.07 ± 0.01	96.52 ± 0.7
	24h	0.11 ± 0.09	3.53 ± 0.6	0.06 ± 0.04	96.32 ± 0.5

Suppl. Table 1. Differentiation potential of sorted CD28$^+$CD57$^-$, DP, DN and CD28$^-$CD57$^+$ CD8$^+$ T cells incubated for 4h, 12h and 24h. Sorted CD28$^+$CD57$^-$, DP, DN and CD28$^-$CD57$^+$ CD8$^+$ T cells were incubated in the presence of 1µg/ml α-CD3 and 10ng/ml IL-2 for 4h, 12h or 24h and stained with anti-CD3, anti-CD8, anti-CD57 and anti-CD28 Abs. For each condition, frequency of CD28$^+$CD57$^-$, DP, DN and CD28$^-$CD57$^+$ CD8$^+$ T cells is shown as mean of 3 samples ± SEM pooled from 3 independent experiments.
